# A novel intelligent global harmony search algorithm based on improved search stability strategy

**DOI:** 10.1038/s41598-023-34736-1

**Published:** 2023-05-12

**Authors:** Jinglin Wang, Haibin Ouyang, Chunliang Zhang, Steven Li, Jianhua Xiang

**Affiliations:** 1 School of Mechanical and Electric Engineering, Guangzhou University, Guangzhou, 510006 China; 2 Graduate School of Business and Law, RMIT University, Melbourne, 3000 Australia

**Keywords:** Applied mathematics, Computational science, Computer science

## Abstract

Harmony search (HS) is a new swarm intelligent algorithm inspired by the process of music improvisation. Over the past decade, HS algorithm has been applied to many practical engineering problems. However, for some complex practical problems, there are some remaining issues such as premature convergence, low optimization accuracy and slow convergence speed. To address these issues, this paper proposes a novel intelligent global harmony search algorithm based on improved search stability strategy (NIGHS). In the search process, NIGHS uses the adaptive mean of harmony memory library to build a stable trust region around the global best harmony, and proposes a new coupling operation based on linear proportional relation, so that the algorithm can adaptively adjust the ability of exploration and exploitation in the search process and avoid premature convergence. In addition, the dynamic Gauss fine-tuning is adopted in the stable trust region to accelerate the convergence speed and improve the optimization accuracy. The common CEC2017 test functions are employed to test the proposed algorithm, the results show that NIGHS algorithm has a faster convergence speed and better optimization accuracy compared to the HS algorithm and its improved versions.

## Introduction

In the past few decades, many meta-heuristic search algorithms have been developed and performed well in solving various optimization problems in engineering fields. Thus, they have attracted a significant amount of attention in recent years.

Many traditional numerical optimization algorithms mainly calculate based on differential and gradient descent principles. Therefore, when solving some complex optimization problems, such as discrete structure optimization^1^, water supply network design^2^ and vehicle routing problem^3^ etc., the traditional optimization algorithm cannot get reasonable and accurate solutions in a limited time. With the continuous development of science and technology, the actual engineering optimization problem becomes more and more complex, the scale of calculation is more and more large, and the constraints are more and more. The defects of traditional optimization algorithms in solving these problems become more and more obvious, so effective and simple meta-heuristic search algorithms become more and more important. Since the meta-heuristic search algorithm is an iterative calculation by simulating the randomness and regularity of some phenomena in nature and life, it does not require too much mathematical form of the problem and has good global search ability, so it is suitable for solving these optimization problems. For example, genetic algorithm (GA) simulates the process of biological evolution and natural selection, establishes selection, crossover and mutation operations according to biological evolution, and simulates the process of biological evolution in iterative calculation^4^. Inspired by the foraging behavior of birds, the particle swarm optimization algorithm (PSO) compares the optimal solution to the problem to food, updates the position of each bird according to the position of the bird closest to the food and each bird's own position, and simulates the process of birds constantly searching for food through iterative calculation^5^. Mirjalili and Lewis proposed whale optimization algorithm (WOA) by simulating the behavior of whale predation on fish and using random or optimal individuals to simulate the hunting behavior of humpback whale^6^. Alsattar simulates the hunting strategy and intelligent social behavior of condor when looking for prey, and proposes the bald eagle search optimization algorithm (BES)^7^. In various optimization problems, these algorithms show better performance than traditional numerical methods in solving optimization solutions.

Harmony search (HS) algorithm is a population-based meta-heuristic search algorithm, which is inspired by the phenomenon that musicians repeatedly adjust the pitch of their instruments to achieve a beautiful harmonic state. It usually has a small number of adjustable parameters and is easy to implement. Based on these advantages, HS has been successfully applied to a large number of optimization problems^8,9^, such as job-shop scheduling^10–12^, function optimization^13–25^, neural network training optimization^26^, network design^27^, Knapsack problem^28^, etc. However, HS algorithm has the disadvantages of slow convergence speed^29^, premature convergence and low optimization accuracy. In order to improve the performance of HS algorithm, four aspects of parameter adjustment, strategy design, algorithm mixing and algorithm application are mainly improved.

The main approaches for parameter adjustment include empirical parameter adjustment, dynamic parameter adjustment and adaptive parameter adjustment. Mahdavi et al. proposed an improved harmony search algorithm (IHS) by dynamically adjusting PAR and BW parameters, and effectively enhanced the search capability of the algorithm^30^. Pan et al. proposed an adaptive global optimal harmony search (SGHS) algorithm, which stores the information in the global optimal scheme and uses this information to adjust the harmony memory consideration rate (HMCR) and pitch adjustment rate (PAR) according to the proposed learning mechanism^20^. Khalili et al. eliminated various parameters that needed to be defined before optimization of the original algorithm, and adjusted parameters HMCR and PAR into dynamic mode. HMCR and PAR increase gradually in the early stage of algorithm calculation, and decrease gradually in the second half^31^. Zhu et al. proposed an improved differential-based harmony search algorithm with linear dynamic domain (ID-HS-LDD). In the ID-HS-LDD algorithm, the improved difference based method is adopted to adjust the value of BW, and the changes of HMCR and PAR are divided into dynamic adjustment and fixed value^32^. Li et al. artificially minimized the thrust of bovine cortical bone VAD, and proposed an information feedback adaptive harmony search (IFSHS) algorithm, which evaluated the optimization parameters and fed back the evaluation information into the dynamic adjustment of the parameters^33^. Li et al. proposed an improved harmonic search algorithm, called the global optimal adaptive harmony search algorithm (AGOHS), which overcomes the problem of traditional HS parameter setting^34^. Mahmoudi et al. adjusted the bandwidth (BW), pitch adjustment rate (PAR) and other parameters of the harmonic search algorithm through the membership function and fuzzy rules designed to improve the performance of the algorithm^35^. Loor et al. proposed an adaptive improved harmony (AIHS) optimization algorithm based on two adjustment stages, which designed new dynamic BW and dynamic HMCR, and used the maximum vector and minimum vector in HM to dynamically adjust BW^36^. Cui et al. dynamically adjusted parameter BW according to the square root of the mean value of the global harmony vector after each update, so as to obtain better fine-tuning performance and convergence speed, and proposed the opposition-based Learning parameter-adjusting Harmony Search (OLPDHS) Algorithm^37^.

Improvements based on the strategy design include the following works. In order to solve the team orientation problem (TOP), Tsakirakis et al. added a new strategy called "similarity process" into the standard HS process. According to the set similarity parameter SP and similarity matrix SM, the solution space is too large, and proposed the similarity mixed harmony search (SHHS) algorithm^38^. Li et al. proposed a novel enhanced harmony search (NEHS) algorithm. The algorithm divides the calculation process into three stages and fine-tuning with three different BW levels to enhance the global and local search capabilities. Meanwhile, the algorithm uses the global best and global worst harmony vectors to update HM in the second half generation of HMC operation^39^. In order to use HS algorithm to better solve the traveling salesman problem, Boryczka and Szwarc designed a mechanism of resetting HM element to improve adjacent solutions in harmonic memory HM, and activated this mechanism when the number of iterations reached the set number R^40^. In order to improve initial harmonic memory, Doush et al. made use of nearest neighbor (NN) and constructively modified NEH, and proposed an improved harmony search algorithm with proximity heuristic(MHSNH). Another adjacent heuristic method is also applied in this algorithm to improve the resulting harmonic vector^41^. Liu et al. proposed a modified Harmony Search (MHS) algorithm with a control strategy, in which the effective mutation operator and crossover operator are used to avoid falling into local optimum, and adaptive relationship is used to improve the flexibility of crossover^42^. The basic HS algorithm is not suitable for large networks, complex constraints and high dimensions. Therefore, Wang et al. proposed the conductor harmony search (CHS) algorithm, which introduced time series constraints in day-ahead optimal scheduling problems into fractional constraints and state matrix, and used the state matrix to record the values of time series constraints^43^. In the improved harmony search algorithm with hybrid convergence mechanism (AHS-HCM) proposed by Zhu and Tang (2021), They introduced a convergence coefficient into the harmonic wave to adjust the optimization performance, and proposed a quadratic nonlinear convergence region for global search, overcoming the disadvantages of low accuracy of basic HS optimization and easy to fall into local optimal^44^. In 2021, Pan et al. proposed an adaptive agent-based harmonious search algorithm (ASBHSA) to artificially accelerate the design and optimization of VSC structures. This algorithm integrated GHS algorithm into the optimization framework, and designed an adaptive sampling method based on the combination of distance-based filling criteria and radial basis function (RBF)^45^. Li et al. proposed a new harmony search algorithm to solve the image segmentation problem. In this algorithm, central harmony and central congestion distance are introduced in the initial harmonic memory stage to reduce the possibility of local aggregation of initial solutions, and a new harmonic generation strategy based on explicit harmonic learning experience is constructed^46^.

In fact, many new meta-heuristics algorithms are based on the combination of existing meta-heuristics algorithms^47^. At present, many scholars make use of the characteristics of existing heuristic algorithms and combine heuristic algorithms with harmony search algorithm to solve some problems existing in traditional harmony search algorithm. For example, Fesanghary et al. proposed the hybrid harmony search (HHS) algorithm, which combines HS algorithm with sequential quadratic programming (SQP) algorithm to improve the local search efficiency and the precision of the algorithm^48^. Wang and Li proposed the co-evolutionary differential evolution with harmony search algorithm (CDEHS). In the first half of the algorithm, the two populations respectively implement the optimization mechanism of differential evolution algorithm (DE) and the optimization mechanism of HS. In the second half of the algorithm, HS population stops evolving and provides the best solution for DE population evolution^49^. In order to improve the convergence ability of the basic harmony search algorithm, Kayabekir et al. proposed a new hybrid harmony search (HHS) algorithm, which combines the local search phase of the harmony search algorithm with the global search phase of the flower pollination algorithm (FPA). Compared with other heuristic algorithms, HHS algorithm has fewer iterations and smaller total potential energy value^50^. Shaikh et al. found that the mixture of HS and SA could expand the search scope and avoid premature convergence of HS, so they proposed the hS-SA algorithm, which has significant search ability in the early stage far away from the nearby optimal value to avoid falling into the local optimal^51^. Zhang et al. proposed an improved harmonious search (IC-HS) algorithm based on clustering, which combined the clustering algorithm when initializing the harmonious memory library, made the initial value distribution more uniform, avoided premature convergence, and achieved faster convergence speed and higher efficiency^52^. In order to avoid falling into local optimization or becoming unstable, Radman et al. combined bidirectional evolutionary structure optimization (BESO) with HS, and utilized the random and random characteristics of HS to reduce the risk of the process falling into local optimization or becoming unstable^53^. Gong et al. combined and complemented harmonious search and Tabu search according to their advantages and disadvantages. The local search capability of HS algorithm is enhanced by combining the advantages of harmony search algorithm in early and middle stage global search with the neighborhood search of Tabu search algorithm^54^. In order to improve the performance of HS algorithm, Amini and Ghaderi introduced pheromone values and heuristic values of ant colony algorithm into HS algorithm, and used them to regenerate probabilistic mass function (PMF), and then used PMF to fill HM, so that HS algorithm could avoid falling into local minimum^55^. Similarly, in order to avoid falling into local minima, Gheisarnejad et al. improved the spawning and migration operations of Cuckoo Search Algorithm (CSA) and added them into HS algorithm to create a new update method^56^.

HS algorithm also has good innovation in many practical applications. Mahmoudi et al. used the improved harmony search algorithm to optimize the loading mode and rationally design the pressurized water reactor core fuel management, which increased the fitness function value by 15.35% and improved the economy and safety of nuclear reactors^35^. Li et al. applied an improved harmony search algorithm to optimize the automatic guided vehicle (AGV) scheduling algorithm. The mathematical model including the total travel distance of AGV and the standard deviation of the wait time of NC material buffer is improved^57^. Gong et al. have improved the layout planning of automated assembly lines and flexible manufacturing production facilities through a new harmony Tabu search algorithm, and have used this algorithm to explore the optimal construction conditions for the mechanical properties of composite particleboard^54^. Szwarc et al. proposed a new harmony search algorithm design method for directional problems. Through experiments, compared with other algorithms, the efficiency of this algorithm is more significant, and the average error is less than 0.01%^58^. In order to optimize the layout of single-camera and multi-lens devices, Wang et al. optimized 3D multiphase flow imaging device through an improved harmony search algorithm, which can be used to measure transparent objects and opaque objects in the center of the 3D observation area^59^. In order to obtain higher accuracy in fake news detection, Huang and Chen proposed an adaptive and improved harmony search algorithm model, which is superior to the existing model and has a maximum accuracy of 99.4%^60^. Yong transforms the general LCP into a nonlinear equation by means of the NCP—function, and uses a new global harmony search algorithm (NGHS) to solve the problem. Numerical results show that this algorithm has a faster convergence rate than other algorithms and overcomes the shortcomings of interior point method^61^. Jeddi et al. proposed a new improved HS algorithm, which solved the established problems of robust dynamic distributed energy (DER) planning, achieved the balance between development and exploration capabilities, and improved the performance of dynamic distributed energy planning in distribution networks^62^. Determining the optimal variable of electricity quantity under the most economical condition is the key to meet the demand of electricity. Therefore, Maleki et al. used the IHS algorithm to determine the optimal size of a hybrid energy system consisting of photovoltaic panels and batteries^63^. In terms of gene selection and classification of high-dimensional medical data, Dash et al. proposed a gene selection method based on adaptive harmony search algorithm to overcome the difficulty of gene selection in large search spatial representation of microarray data^64^. Botella Langa et al. applied the harmonious search algorithm to the optimal design of urban water distribution network, and the experiment showed that compared with other heuristic algorithms, the harmony search algorithm could obtain more convenient solutions at more reasonable computational costs^65^. The type of solar panel has a big impact on the optimal size of a hybrid photovoltaic cell solution. Therefore, Liu,J et al. proposed a new global dynamic harmony search algorithm to solve the optimal size of hybrid photovoltaic cell schemes^66^. Due to the outbreak of COVID-19 in recent years, there is a shortage of international nurses, and it is difficult to provide convenient roster for nurses. The problem of nurse roster is a well-known non-deterministic polynomial time difficult combinatorial optimization problem. Therefore, Mohammed Hadwan proposed an annealing harmony search algorithm (AHSA) to solve this problem^67^. In medicine and genetics, Shouheng Tuo et al. developed and applied some excellent modified harmony search algorithm to detect higher-order SNP epistatic interactions. Through experimental comparison, it is proved that this algorithm can effectively perform multiple high-order detection tasks for high-order epistatic interaction, and improve the recognition ability of different epistatic models^68–70^.

In the HS algorithm, the HM library is updated by comparing whether the new harmonies produced in each generation are better than the worst harmonies in the HM library. This means that HM library can actually be regarded as the preservation library of the global best harmonies in history, storing the historical information of the best harmonies in the past. To date, a lot of research has been done on the parameter adjustment of the algorithm and the design of new harmony generation strategy, but little research has been done on how to use all the historical information in HS library to assist the generation of new harmony. Studying how to utilize the historical information in HM library is still an important and significant research area. At the same time, there are little research on how to use the relationship between individual values of different dimensions in the algorithm calculation.This research aims to make some contribution in these two aspects.

In this paper, we propose an improved version of the Harmony Search (HS) algorithm. This algorithm is also an improvement over the Intelligent Global Harmony Search (IGHS) algorithm proposed by Ehsan Valian et al. in 2014^71^. We adopted the trust region and simple coupling strategy and named our algorithm the Novel Intelligent Harmony Search (NIGHS) algorithm. The NIGHS algorithm has the following features:

1. Construct a stable trust region based on all the historical information in the HM library. For each variable, the trust region is constructed with the mean value of the variable in HM library as the boundary and the global best harmony as the center.
2. Dynamic Gauss fine-tuning in the trust region to enhance local search ability. Gaussian fine-tuning with dynamic adjustment of step size with the new solution obtained randomly in the trust region improves the convergence speed and accuracy of the algorithm.
3. Improved coupling operation between different dimensions. The relationship between other dimensions and the current dimension is used to adjust the value of the current dimension, which provides a large reasonable fluctuation for the dimension in the early stage of the algorithm and enhances the ability to get rid of the local optimal.

The proposed NIGHS is compared with the basic version of harmony search (HS)^72^, the improved harmony search algorithm (IHS)^30^, the geometric Harmony search algorithm (GHS)^73^, the novel global harmony search algorithm (NGHS)^20^, the self-adaptive global best harmony search algorithm(SGHS)^74^, the intelligent global harmony search algorithm (IGHS) which was proposed by Ehsan Valian et al. in 2014^71^ and the intersect mutation global harmony search algorithm (IMGHS)^75^. These methods are tested and evaluated using CEC 2017 benchmark functions. Our experimental results reveal that the proposed NIGHS algorithm is superior to the comparison HS variants on many benchmark functions and has a more robust convergence when optimizing objective functions in terms of the solution accuracy and efficiency.

The remainder of this paper is organized as follows: “Preliminaries” section provides the background information of harmony search (HS), novel global harmony search (NGHS), and intelligent global harmony search algorithm (IGHS). “The proposed novel intelligent global harmony search algorithm (NIGHS)” section describes the proposed novel intelligent Global Harmony search algorithm (NIGHS). The experimental results and analysis are presented in “Experimental results and analysis” section. The concluding remarks are given in “Conclusions” section.

## Preliminaries

### Harmony search (HS) algorithm

Harmony search (HS) is a type of population-based algorithm that was first introduced by Geem, Z. W. (2001). The HS algorithm is inspired by the improvisation of music players, and its main steps are as follows.

*Step 1: Initialization of parameters* In this step, we define the values of each parameter of HS. Among them, these parameters include the dimension of the variable (N), the lower and upper limits of the search domain (LB,UB), harmonization memory bank size (HMS), harmonization memory consideration rate (HMCR), pitch adjustment rate (PAR), bandwidth vector (BW), and maximum number of iterations (NI).

*Step 2: Initialization of the harmony memory* The harmonic memory bank (HM) is an N × HMS matrix, whose initial value consists of N vectors randomly generated between the lower and upper bounds (LB, UB) of the problem search domain. The formula is as follows:

1 $${\text{x}}_{{{\text{i}},{\text{j}}}} = {\text{LB}}_{{\text{j}}} + \left( {{\text{UB}}_{{\text{j}}} - {\text{LB}}_{{\text{j}}} } \right){\text{*rand}}\left[ {0,1} \right].$$

2 $${\text{HM}} = [{\text{x}}_{1} ,{\text{x}}_{2} ,{\text{x}}_{3} , \ldots {\text{x}}_{{{\text{HMS}}}} ]^{{\text{T}}}$$

where i = 1,2……,HMS, and j = 1,2,…,N.

*Step 3: Improvisation of a new harmony* In this step, a new harmony ($x^{new}$) is generated based on:harmony memory consideration rate (HMCR), pitch adjusting rate (PAR) and a random choice as described below. :

*Step 3.1: Harmony memory consideration rate (HMCR)* First, a new random number ${\text{r}}1 \in \left( {0,1} \right)$ is generated. Then, it is compared with the harmony memory consideration rate (HMCR). If ${\text{r}}1 < {\text{HMCR}}$, each component of the new harmony is chosen randomly from the harmony memory (HM) components according to

3 $$x_{j}^{new} = x_{j}^{a}$$

where ${\text{j}} = 1,2, \ldots ,{\text{N}}$,and $x^{a}$ is a random harmony vectors from harmony memory (HM).

*Step 3.2: Pitch adjusting rate (PAR)* A new random number ${\text{r}}2 \in \left( {0,1} \right)$ is generated, and if ${\text{r}}2 < {\text{PAR}}$, the component selected in the harmonic memory Consideration rate (HMCR) step is fine-tuned according to bandwidth BW, the formula is as follows:

4 $$x_{j}^{new} = \left\{ {\begin{array}{*{20}l} {x_{j}^{new} \pm rand \times BW} \hfill & {if\; r2 < PAR} \hfill \\ {x_{j}^{new} } \hfill & {otherwise} \hfill \\ \end{array} } \right.$$

where $${\text{j}} = 1,2, \ldots ,{\text{N}}$$.

*Step 3.3: Random generation* Each component that is not selected for the harmonious Memory Consideration Rate (HMCR) will be replaced with a value randomly generated in the search field, namely:

5 $$x_{j}^{new} = {\text{LB}}_{{\text{j}}} + \left( {{\text{UB}}_{{\text{j}}} - {\text{LB}}_{{\text{j}}} } \right){\text{*rand}}\left[ {0,1} \right].$$

where $${\text{j}} = 1,2, \ldots ,{\text{N}}$$.

*Step 4: Harmony memory (HM) update* Here a new harmony is generated. If the fitness value of the new harmony (${\text{x}}^{{{\text{new}}}}$) is better than the fitness value of the worst harmony (${\text{x}}^{{{\text{worst}}}}$) in HM, the worst harmony in HM will be replaced by the new harmony.

*Step 5: Check the termination criterion* If the number of the current iteration (t) is less than the maximum number of iterations (NI), then Steps 3 and 4 are repeated. Otherwise, the optimization process stops.

The HS algorithm are shown in Algorithm 1.
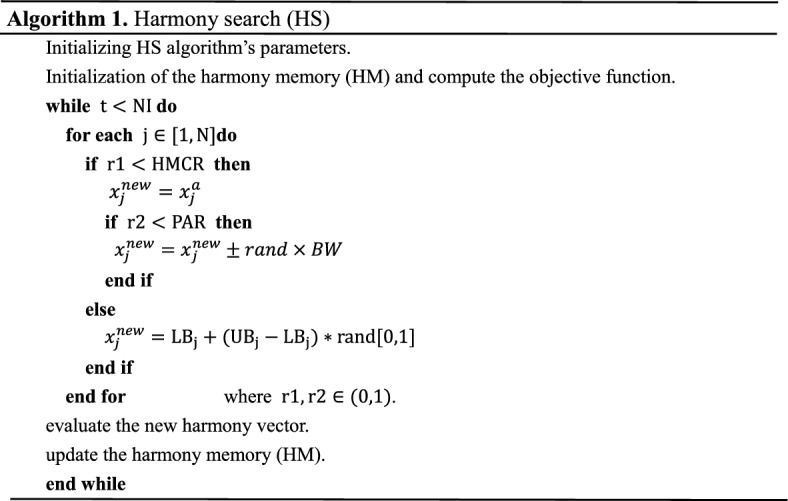


### Novel global harmony search (NGHS)

The novel global harmony search (NGHS) algorithm^74^ is a global type of harmony search (HS) whose genetic mutation operator is applied after updating its position to escape from the local minima. It modifies the improvisation step of the HS by substituting HMCR and PAR based on the genetic mutation probability $P_{m}$. The main steps of NGHS algorithm are shown in Algorithm 2.
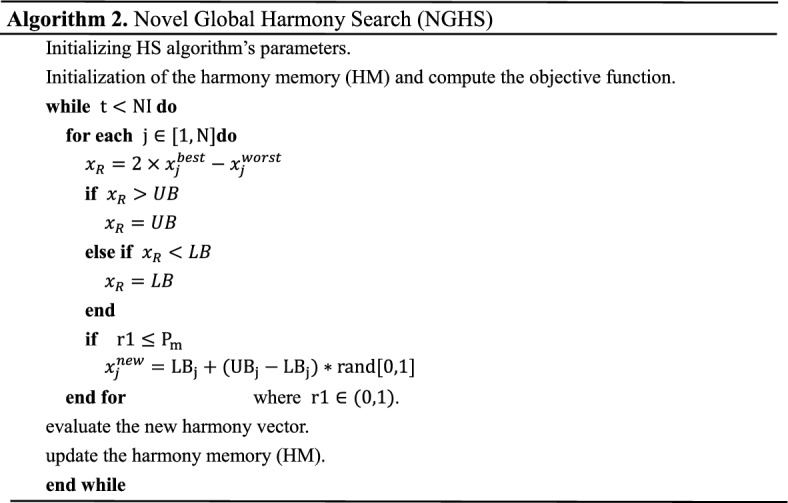


### Intelligent global harmony search (IGHS)

The intelligent global harmony search (IGHS) algorithm^71^ is a variant of novel global harmony search (NGHS). It modifies the improvisation step of the NGHS in such a way that the new harmony imitates one dimension of the best harmony in the HM. The main steps of IGHS algorithm are shown in Algorithm 3.
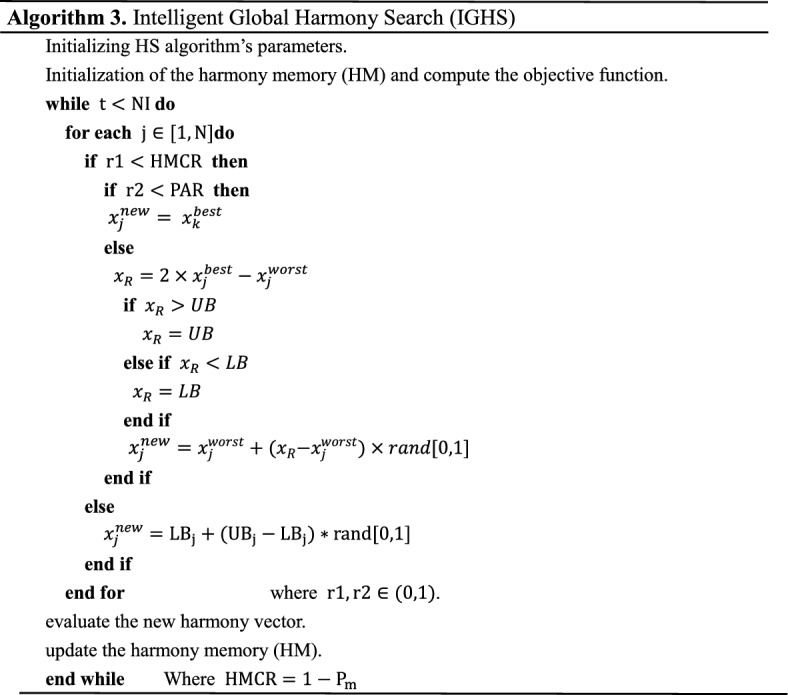


## The proposed novel intelligent global harmony search algorithm (NIGHS)

In this section, we will introduce an improved version of HS algorithm, namely the novel intelligent global harmony search algorithm (NIGHS). The main improvement in the NIGHS are the stable trust region and improved coupling operation.

### Stable trust region

**Figure 1:**
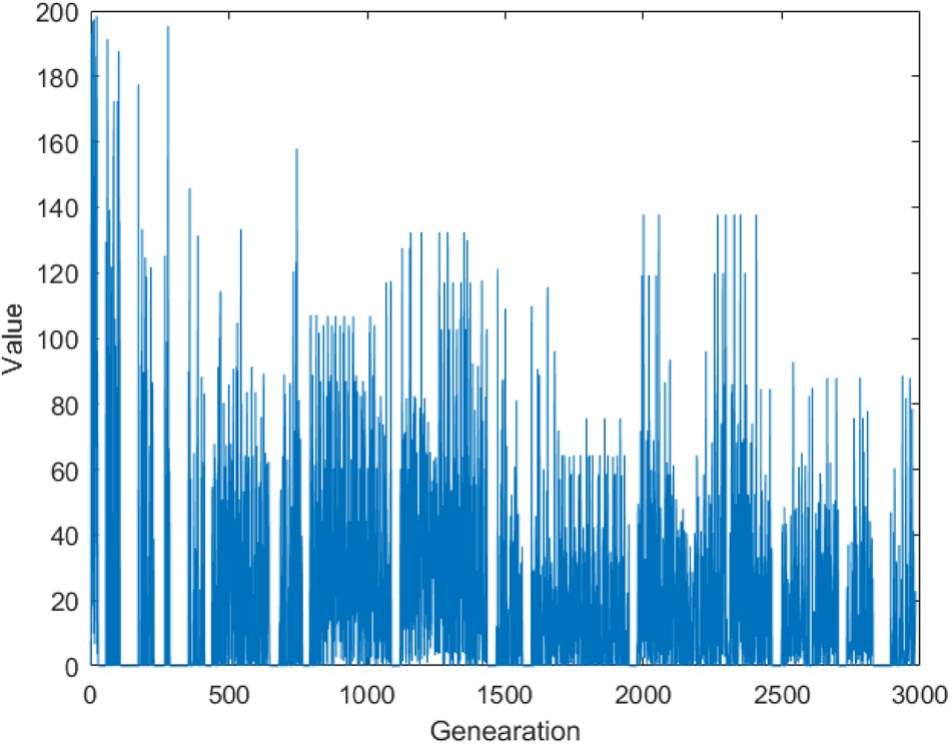
The width of trust region in IGHS.

According to the HS algorithm, each variable of the new harmony in each generation is generated by fine-tuning the variable of the same dimension of any harmony in the HM library with a certain probability, or randomly generated globally. The multiple harmonies in the HM library provide the diversity for the generation of new harmonies. In the NGHS algorithm, it is changed to adjusting the global best harmony search position instead of replacing the original selection operation. The IGHS Algorithm adds coupling operation between variables of different dimensions (Algorithm 3, Line 7) on the basis of the global adjustment strategy of NGHS, which increases the diversity of population. The adjustment range of the global best harmony search is only determined by the best and worst harmonies of the HM library, and the range of the trust region is unstable, and the convergence is poor. In IGHS algorithm, the width of trust region is easy to be zero, which leads to the stagnation of iterative search, as shown in Fig. 1.

**Figure 2:**
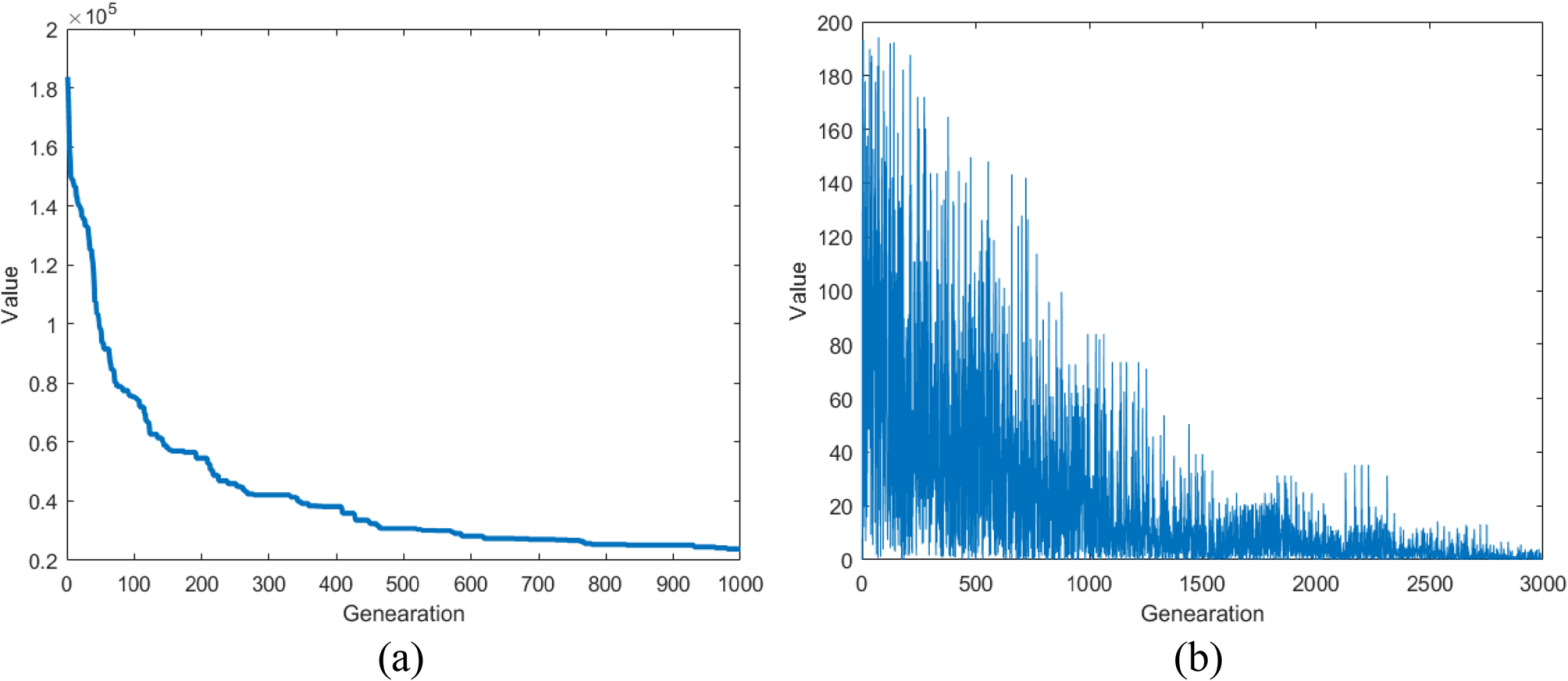
**a** The value of the mean of HM libraries in HS and **b** the width of trust region in NIGHS.

Therefore, how to build a stable trust region is the key to improve the optimization ability of the IGHS algorithm. With the iteration of HM library, the harmony of HM library will gradually approach to the global optimal harmony. The iterative change of the fitness mean of HM library is shown in Fig. 2a. It is natural to think that a stable trust region can be constructed through the mean and best harmony of the HM library. The trust region is constructed as follows:

6 $$x_{j}^{mean} = \frac{{\mathop \sum \nolimits_{i = 1}^{HMS} x_{i,j} }}{HMS}$$

7 $$x_{R} = 2 \times x_{j}^{best} - x_{j}^{mean}$$

**Figure 3:**
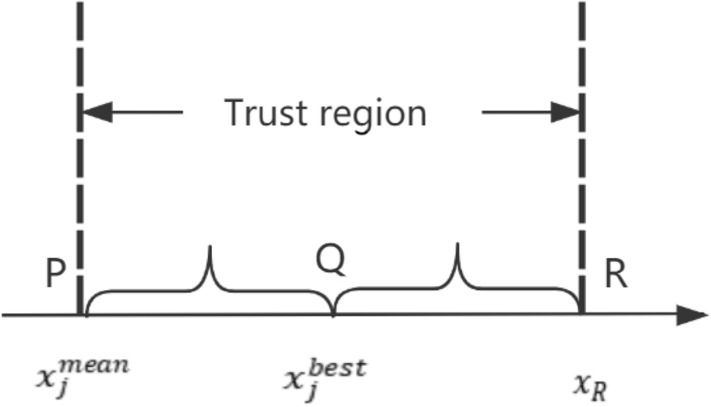
The schematic diagram of position updating.

Here, HMS represents the size of HM library, and ${\text{x}}_{{\text{j}}}^{{{\text{mean}}}}$ represents the average value of this variable in the HM library. The boundary treatment method is adopted, if the ${\text{x}}_{{\text{R}}}$ exceeds the value range of [LB,UB]. The schematic diagram of the trust region is shown in Fig. 3. We can see that the trust region is centered on the best harmony and its width is twice the distance between ${\text{x}}_{{\text{j}}}^{{{\text{mean}}}}$ and ${\text{x}}_{{\text{j}}}^{{{\text{best}}}}$, under the premise that it does not exceed the search range. In the early stage of the algorithm, the trust region is wide, so that the algorithm can conduct global search and avoid premature convergence. With the progress of iteration, the trust region is gradually reduced, the convergence speed is accelerated, and the solution accuracy is also becoming higher and higher. The width of the trust region varies over iterations as shown in Fig. 2b.

**Figure 4:**
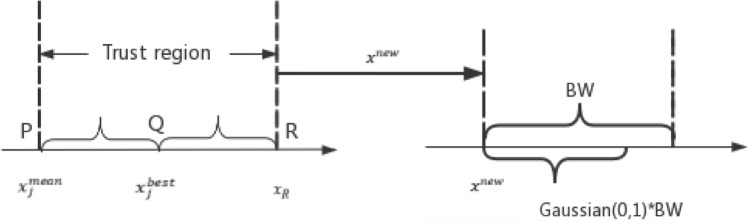
The schematic diagram of secondary search.

**Figure 5:**
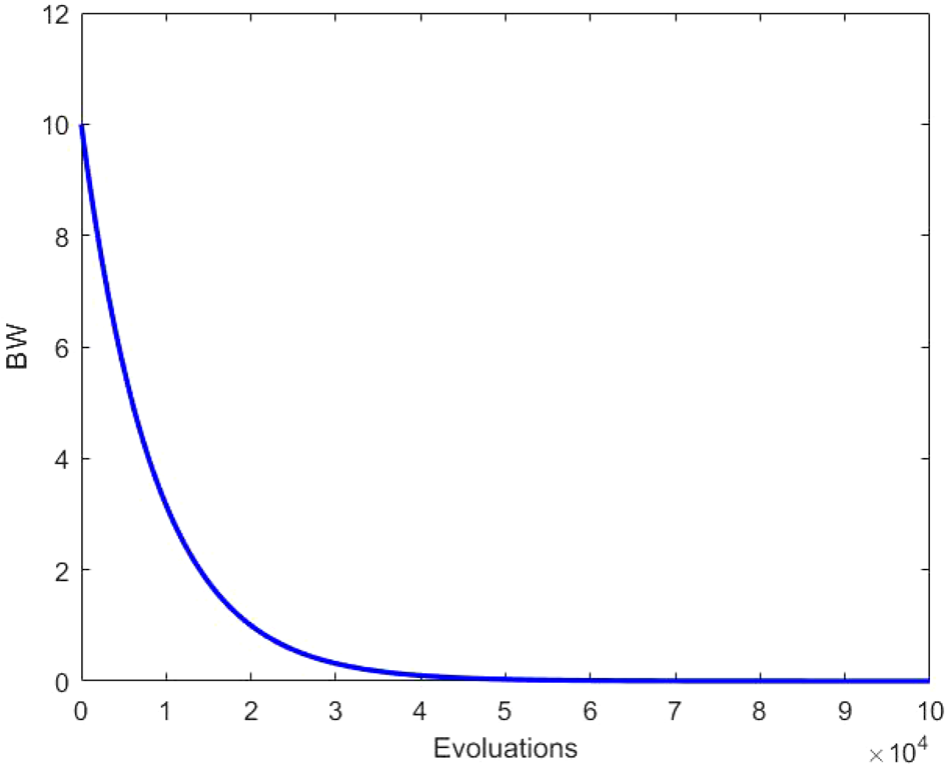
The dynamic change of BW.

To enhance the local search ability, we conduct a Gaussian fine-tuning of the selected value after a random search in the trust region. The process principle is shown in Fig. 4, and the dynamic change of BW is shown in Fig. 5. The specific steps are as follows:

8 $$x_{j}^{new} = x_{j}^{mean} + \left( {x_{R} - x_{j}^{mean} } \right) \times rand\left[ {0,1} \right]$$

9 $$BW = BW_{max} *exp\left( {c*\frac{t}{NI}} \right)$$

10 $$c = ln\left( {\frac{{BW_{min} }}{{BW_{max} }}} \right)$$

11 $$x_{j}^{new} = x_{j}^{new} + Gaussian\left( {0,1} \right)*BW$$

where the $Gaussian\left( {0,1} \right)$ represents a Gaussian distribution random number with a mean value of 0 and a standard deviation of 1.

### Improved coupling operation

In the IGHS algorithm, a simple coupling operation, ${\text{x}}_{{\text{j}}}^{{{\text{new}}}} =$ ${\text{x}}_{{\text{k}}}^{{{\text{best}}}}$, is adopted, and the value of other dimensions is randomly selected to replace the value of this dimension in the best harmony. When the dimensions of the optimal solution of the problem are the same, the strategy will have a good effect. But the optimal values of each dimension are different in practical engineering problems. Therefore, we improved the strategy:

12 $$x_{j}^{new} = \left( {0.6*\frac{{x_{j}^{best} }}{{x_{k}^{best} }} + 0.4*\frac{{x_{j}^{worst} }}{{x_{k}^{worst} }}} \right)*x_{k}^{mean}$$

We use the linear proportional relationship between any two dimensions of the best harmony and the worst harmony according to a certain weight combination to form a new linear proportional relationship, in the mean harmony according to the linear relationship to generate new values. This strategy is not a simple direct replacement like IGHS, but a reasonable generation of new values according to the linear relationship between dimensions, which can improve the shortcomings of the coupling operation of IGHS mentioned above. In order for the algorithm to do this at the right time at runtime, we dynamically adjust the HMCR and PAR:

13 $$HMCR = 0.85 + 0.3*\sqrt { \frac{{\left( {it - 1} \right)}}{{\left( {NI - 1} \right)}}*\left( {1 - \frac{{\left( {it - 1} \right)}}{{\left( {NI - 1} \right)}}} \right)}$$

14 $$PAR = PAR_{max} - \left( {PAR_{max} - PAR_{min} } \right)*\frac{t}{NI}$$

**Figure 6:**
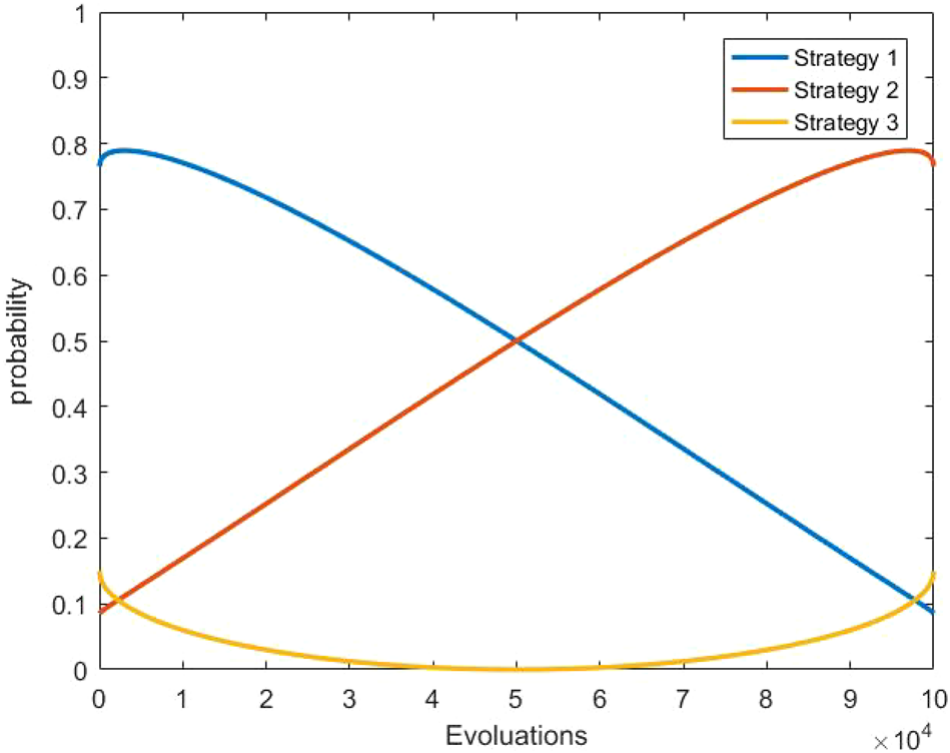
The probability change for each strategy.

The dynamic tuning of HMCR and PAR allows the algorithm to select the right operation with a high probability at the right time. The plotted probabilities of each operation at different time periods are shown in Fig. 6, where strategy 1 is the improved coupling operation, strategy 2 is the stable trust region search, and strategy 3 is the global random search.

In the early stage of algorithm operation, strategy 1 has the highest probability, and a wide range of search is carried out through coupling operation to avoid premature convergence, and the probability gradually decreases. The probability of strategy 2 gradually increases with the running of the algorithm, to accelerate the convergence speed and improve the convergence accuracy through the stable trust region search and Gaussian fine tuning. Meanwhile, the global random search of strategy 3 maintains a certain probability in the late stage of the algorithm, which provides a certain opportunity to escape from the local optimal and find a better one.

### The steps of the NIGHS algorithm

The complete steps of NIGHS are as follows:

*Step 1: Initialization of NIGHS parameters* In this step, the IGHS algorithm parameters are defined, such as the number of decision variables (N), lower and upper bounds (LB,UB), harmony memory size (HMS), harmony memory consideration rate (HMCR), pitch adjusting rate (PAR), and maximum number of iterations(NI).

*Step 2: Initialization of the harmony memory (HM)* First, the harmony memory is randomly placed between the upper and lower bounds (LB,UB) of optimization problems according to Eqs. (1) and (2).

*Step 3* Update the HMCR, PAR and BW. In this step we update the HMCR, PAR and BW through the Eqs. (9), (10), (13) and (14).

*Step 4: Improvisation of a new harmony* In this step, the new harmony (${\text{x}}^{{{\text{new}}}}$) is created by means of Algorithm 4 (Lines 6–21).We replace ${\text{x}}^{{{\text{worst}}}}$ with ${\text{x}}^{{{\text{mean}}}}$, generate the trust region between ${\text{x}}_{{\text{R}}}$ and ${\text{x}}^{{{\text{mean}}}}$(Lines 6–14), and add the Gaussian fine-tuning strategy (Line 15).

*Step 5: Harmony memory (HM) update* Here a new harmony is generated. If the fitness value of the new harmony (${\text{x}}^{{{\text{new}}}}$) is better than the fitness value of the worst harmony (${\text{x}}^{{{\text{worst}}}}$) in HM, the worst harmony in HM will be replaced by the new harmony.

*Step 6: Check the termination criterion* If the number of the current iteration (t) is less than the maximum number of iterations (NI), then Steps 3 and 4 are repeated. Otherwise, the optimization process stops.

**Figure 7:**
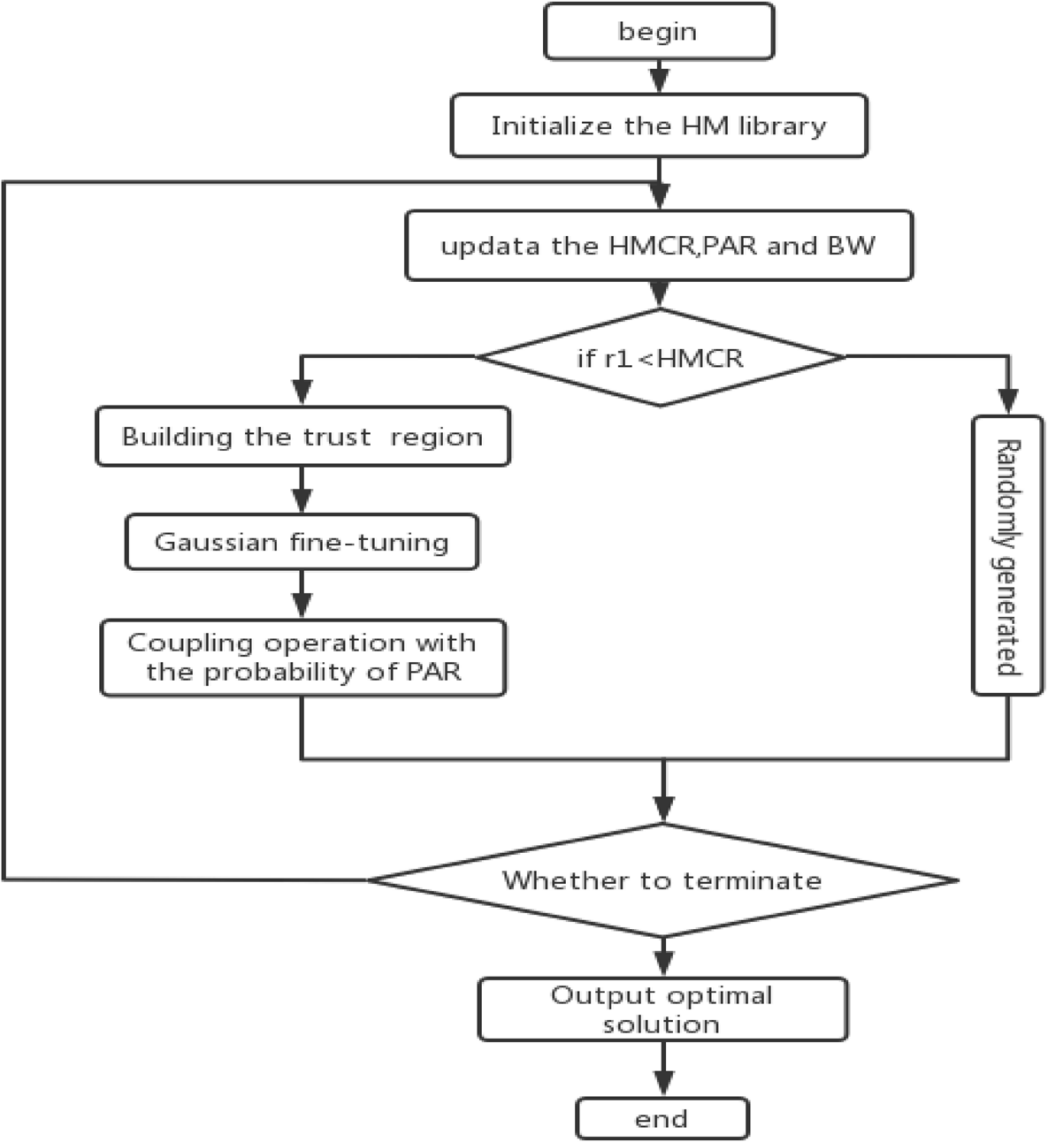
The overall flow chart of the NIGHS algorithm.

The NIGHS algorithm are shown in Algorithm 4 and the flow chart of NIGHS algorithm is given in Fig. 7.

## Experimental results and analysis

### Computing environment and parameter settings

In this research work, we use the MATLAB software to implement the program to search for the trial solutions. The solution-finding equipment comprised an Intel Core (TM) i5-10300H (2.50 GHz) CPU, 16 GB of memory, and Windows 10 home edition (64-bit) OS. The settings of these algorithms are extracted from Geem et al.^72^, Mahdavi et al.^30^, Degertekin et al.^73^, Zou et al.^74^, Pan.^20^, Valian^71^, Gholam^75^ and are as follows:

HS: HMS = 5, HMCR = 0.9, PAR = 0.3, BW = 0.01;

IHS: HMS = 5, HMCR = 0.9, ${\text{PAR}}_{{{\text{min}}}} = 0.01,{\text{PAR}}_{{{\text{max}}}} = 0.99$,${\text{BW}}_{{{\text{min}}}} = 0.0001$,${\text{BW}}_{{{\text{max}}}} = \frac{{{\text{x}}_{{\text{u}}} - {\text{x}}_{{\text{l}}} }}{20}$;

GHS: HMS = 5, HMCR = 0.9, ${\text{PAR}}_{{{\text{min}}}} = 0.01,{\text{ PAR}}_{{{\text{max}}}} = 0.99$;

SGHS: HMS = 5, HMCRm = 0.98, PARm = 0.9, ${\text{BW}}_{{{\text{min}}}} = 0.0005$, ${\text{BW}}_{{{\text{max}}}} = \frac{{{\text{x}}_{{\text{u}}} - {\text{x}}_{{\text{l}}} }}{10}$,LP = 100;

NGHS: HMS = 5, $${\text{P}}_{{\text{m}}} = 0.005;$$

IGHS: HMS = 5, HMCR = 0.9950, PAR = 0.4;

IMGHS: HMS = 5, HMCR = 0.9, PAR = 0.3, BW = 0.01,${\text{P}}_{{\text{m}}} = 0.005$, ${\upmu }1 = 0.7$, ${\upmu }2 = 0.3$;

NIGHS: HMS = 5, $${\text{PAR}}_{{{\text{min}}}} = 0.1,{\text{PAR}}_{{{\text{max}}}} = 0.9$$, $${\text{BW}}_{{{\text{min}}}} = 0.0001$$, $${\text{BW}}_{{{\text{max}}}} = \frac{{{\text{x}}_{{\text{u}}} - {\text{x}}_{{\text{l}}} }}{20}$$;;

### Benchmark optimization problems

The CEC2017 test set contains 30 numerical minimization test functions, divided into four groups. The first group contains three unimodal functions (f1–f3), the second group contains seven simple multimodal functions (f4–f10), the third group contains ten hybrid functions (f11–f20), and finally the fourth group contains ten composition functions (f21–f30). The global optimal solution of each function is randomly shifted in $\left[ { - 80,80} \right]^{{\text{D}}}$ and given a different rotation matrix. Consider that in the real-world problem, there is very little correlation between all the variables. In CEC2017, variables are randomly divided into child components. The rotation matrix of each subcomponent are generated from standard normally distributed entries by Gram-Schmidt ortho-normalization with condition number c that is equal to 1 or 2. Table 1 summarizes these capabilities and their features. For details on these features, see^76^.

**Table 1:** Benchmark functions of CEC2017.

Function type	No	Function name	Optimal value
Unimodal	$f_{1}$	Shifted and Rotated Bent Cigar Function	100
	$f_{2}$	Shifted and Rotated Sum of Different Power Function	200
	$f_{3}$	Shifted and Rotated Zakharov Function	300
Simple multimodal	$f_{4}$	Shifted and Rotated Rosenbrock’s Function	400
	$f_{5}$	Shifted and Rotated Rastrigin’s Function	500
	$f_{6}$	Shifted and Rotated Expanded Scaffer’s F6 Function	600
	$f_{7}$	Shifted and Rotated Lunacek Bi_Rastrigin	700
	$f_{8}$	Shifted and Rotated Non-Continuous Rastrigin’s Function	800
	$f_{9}$	Shifted and Rotated Levy Function	900
	$f_{10}$	Shifted and Rotated Schwefel’s Function	1000
Hybrid	$f_{11}$	Hybrid function 1 (N = 3)	1100
	$f_{12}$	Hybrid function 2 (N = 3)	1200
	$f_{13}$	Hybrid function 3 (N = 3)	1300
	$f_{14}$	Hybrid function 4 (N = 4)	1400
	$f_{15}$	Hybrid function 5 (N = 4)	1500
	$f_{16}$	Hybrid function 6 (N = 4)	1600
	$f_{17}$	Hybrid function 6 (N = 5)	1700
	$f_{18}$	Hybrid function 6 (N = 5)	1800
	$f_{19}$	Hybrid function 6 (N = 5)	1900
	$f_{20}$	Hybrid function 6 (N = 6)	2000
Composition	$f_{21}$	Composition Function 1 (N = 3)	2100
	$f_{22}$	Composition Function 2 (N = 3)	2200
	$f_{23}$	Composition Function 3 (N = 4)	2300
	$f_{24}$	Composition Function 4 (N = 4)	2400
	$f_{25}$	Composition Function 5 (N = 5)	2500
	$f_{26}$	Composition Function 6 (N = 5)	2600
	$f_{27}$	Composition Function 7 (N = 6)	2700
	$f_{28}$	Composition Function 8 (N = 6)	2800
	$f_{29}$	Composition Function 9 (N = 3)	2900
	$f_{30}$	Composition Function 10 (N = 3)	3000
Search range: $\left[ { - 100,100} \right]^{D}$			

We conducted 51 independent runs of each algorithm on the benchmark function of CEC 2017 test set, respectively set to 10 (10D), 30 (30D) and 50 (50D) dimensions, where the search range of all test functions is [− 100,100]. According to the benchmark rule, the maximum number of function evaluations is set to 10,000 × D, and the error value obtained is zero if it is less than 10 − 8. Finally, we calculate the best value, worst value, mean value and standard deviation (SD) of the obtained data and make a good arrangement.
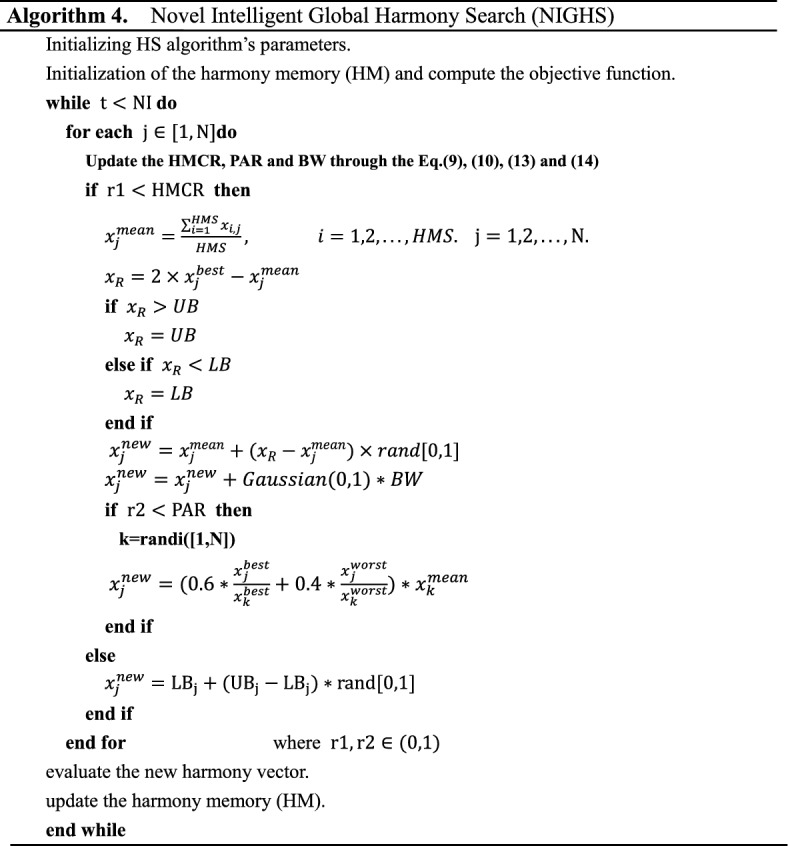


### Experimental results and analysis

According to the famous No Free Lunch Theorem (NFL)^77^, it is impossible to design an algorithm that outperforms all other algorithms for all problems. Hence, we do not expect the proposed NIGHS algorithm is better than all other algorithms for all problems. In this paper, benchmark functions of CEC 2017 are selected to test the algorithm and to see if the NIGHS algorithm performs better than other algorithms on most of the functions.

In this paper, the experiments are conducted with dimension size of 10, 30, and 50, respectively. In the tables below, "+" means that the effect is better than NIGHS algorithm, "-" means that the effect is worse than NIGHS algorithm, and "≈" means that the effect is similar to NIGHS algorithm, that is, the difference between the two is considered to be within 0.1 under the same order of magnitude. The best results are highlighted in bold. Each algorithm is separately run for 51 times under each of the dimension size, with the maximum number of iterations set to 100,000, 300,000 and 500,000. In order to facilitate uniform comparison, we subtract the corresponding optimal value from the result of each calculation of each function and return the optimal value to zero. The best value, worst value, mean value and standard deviation (SD) of the 51 iterations are obtained and reported in Tables 2, 3, 4, 5, 6, 7, 8, 9, 10, 11, 12 and 13. Among them, Tables 2, 3, 4 and 5 presents the comparison results on the best value, worst value, mean value and standard deviation (SD) with dimension size of 10, respectively. Similarly, Tables 6, 7, 8 and 9 presents the comparison results with dimension size of 30, and Tables 10, 11, 12 and 13 presents the comparison results with dimension size of 50.

**Table 2:** The comparison results of the algorithms with D = 10 on the best value.

	HS	IHS	GHS	SGHS	NGHS	IGHS	IMGHS	NIGHS
F1	**8.741654E−02**	6.612149E+00	1.225979E+05	1.968327E+08	4.468254E+04	1.074946E+07	6.464612E+01	1.556800E+00
F2	3.632947E−06	**4.115017E**−**07**	1.559486E+02	6.512164E+05	2.712668E−01	1.072134E+03	4.671981E−05	7.642900E−07
F3	2.089982E−06	**0.000000E**+**00**	9.061780E+01	3.207978E+03	1.533209E+03	2.507052E+01	4.688428E−05	**0.000000E**+**00**
F4	3.710754E−03	2.576605E−03	9.944952E−01	6.277924E+01	2.525647E−01	6.101383E+00	6.431800E−03	**5.557400E**−**04**
F5	2.984877E+00	2.984877E+00	4.285667E+00	4.180563E+01	5.982656E+00	4.129695E+00	9.949610E+00	**1.039200E**+**00**
F6	6.011832E−03	**4.279935E**−**05**	2.656330E−01	2.248821E+01	5.577614E−04	1.281155E+00	4.131947E−04	8.122600E−05
F7	**1.257084E**+**01**	1.694327E+01	1.676604E+01	7.485247E+01	1.893401E+01	2.005186E+01	1.701710E+01	1.603700E+01
F8	**2.984877E**+**00**	3.979836E+00	4.367345E+00	4.732613E+01	8.014882E+00	5.486041E+00	1.392943E+01	3.979800E+00
F9	**0.000000E**+**00**	**0.000000E**+**00**	3.336170E−01	4.409866E+02	5.232141E−01	9.000576E+00	2.818085E+00	**0.000000E**+**00**
F10	**1.524449E**+**01**	1.853454E+01	5.636199E+01	9.719564E+02	1.326838E+02	5.555588E+01	3.620830E+02	1.193900E+02
F11	2.652949E+00	1.628267E+00	5.981441E+00	1.069425E+02	3.111905E+00	7.485135E+00	1.993670E+00	**1.109700E**+**00**
F12	1.095396E+03	**5.180175E**+**02**	1.241866E+04	9.371992E+06	3.406238E+04	1.313379E+04	5.584495E+02	1.068400E+03
F13	7.690055E+01	**1.511266E**+**01**	1.139630E+02	1.521475E+04	9.661038E+01	4.139191E+01	1.528337E+02	1.920000E+01
F14	1.581123E+01	6.299370E+00	2.914410E+01	7.042663E+01	6.475443E+01	1.229702E+01	2.582075E+01	**1.568500E**+**00**
F15	1.850358E+01	7.531623E+00	7.472313E+01	5.044824E+02	1.705654E+01	1.414089E+01	3.991425E+00	**2.110100E**+**00**
F16	**2.525042E**−**02**	2.552894E−01	1.172777E+00	6.838711E+01	1.815747E+00	2.236937E+00	4.727025E−01	6.293800E−01
F17	**1.223648E**−**01**	3.324498E−01	1.966633E+00	6.177889E+01	6.520644E−01	4.413655E+00	3.661098E+00	9.173100E−01
F18	1.471098E+02	2.148435E+02	2.950670E+02	1.561565E+04	2.742366E+02	**5.197683E**+**01**	1.064673E+02	1.015800E+03
F19	**1.290047E**+**00**	1.297368E+00	6.202422E+01	1.560026E+02	6.244870E+01	1.141285E+01	1.207667E+01	2.690800E+00
F20	**2.397760E**−**04**	6.533210E−02	3.325336E+00	7.143468E+01	9.346522E−01	1.417152E+00	9.978029E−01	3.136700E−01
F21	1.010526E+02	1.000000E+02	1.121590E+02	1.101169E+02	1.053501E+02	1.025659E+02	1.000003E+02	**1.000000E**+**02**
F22	3.998117E+01	1.008165E+02	**1.214063E**+**01**	1.057929E+02	8.169584E+01	3.713601E+01	4.310660E+01	1.000000E+02
F23	3.079996E+02	3.064528E+02	3.089794E+02	**1.931382E**+**02**	3.135161E+02	3.066860E+02	3.082938E+02	3.048700E+02
F24	1.000001E+02	1.000002E+02	1.159710E+02	2.095075E+02	1.015804E+02	1.267920E+02	1.000063E+02	**1.000000E**+**02**
F25	3.979291E+02	3.979291E+02	2.673362E+02	4.416780E+02	2.282871E+02	4.177643E+02	**2.002631E**+**02**	3.978200E+02
F26	2.219889E−03	**1.483232E**−**03**	2.015008E+01	4.675250E+02	3.802924E+00	5.314070E+01	2.000598E+02	1.831700E−03
F27	3.892997E+02	3.897899E+02	**3.889627E**+**02**	4.110654E+02	3.960546E+02	3.942888E+02	3.980542E+02	3.923500E+02
F28	3.000004E+02	3.000003E+02	3.181304E+02	3.865826E+02	3.008387E+02	3.195921E+02	3.000062E+02	**3.000000E**+**02**
F29	2.446472E+02	**2.347812E**+**02**	2.415016E+02	3.252426E+02	2.709355E+02	2.479339E+02	2.518119E+02	2.372900E+02
F30	1.825302E+03	2.242530E+03	**1.128505E**+**03**	8.305452E+04	3.202637E+03	2.389064E+04	1.368195E+03	1.575500E+03
+\−\≈	18\10\2	15\9\6	24\6\0	29\1\0	26\4\0	27\3\0	24\6\0	

Significant values are in [bold].

**Table 3:** The comparison results of the algorithms with D = 10 on the worst value.

	HS	IHS	GHS	SGHS	NGHS	IGHS	IMGHS	NIGHS
F1	1.231832E+04	**1.090169E**+**04**	1.038254E+07	4.093758E+09	4.208001E+06	9.586893E+08	1.124221E+04	1.263500E+04
F2	2.625019E+02	5.664594E−04	8.560951E+05	3.398192E+09	4.651870E+07	2.297814E+08	1.640024E−02	**7.057900E**−**05**
F3	1.138191E+03	**0.000000E**+**00**	1.531837E+03	1.281439E+04	7.004171E+04	1.008941E+03	4.157738E−03	1.007500E−07
F4	6.884503E+01	2.904373E+00	1.368941E+02	3.075408E+02	1.340713E+02	1.649587E+02	7.061432E+01	**7.525800E**−**02**
F5	**1.989916E**+**01**	2.487393E+01	2.795900E+01	8.026095E+01	4.899875E+01	4.748891E+01	5.173770E+01	3.183900E+01
F6	2.745874E−01	**1.773854E**−**04**	1.992980E+00	4.579928E+01	1.355952E+00	1.122363E+01	2.938838E+00	8.120000E−04
F7	4.531029E+01	**4.143209E**+**01**	5.263342E+01	1.940923E+02	6.367052E+01	9.848080E+01	6.372838E+01	5.984700E+01
F8	2.487393E+01	2.388530E+01	**2.268459E**+**01**	8.053728E+01	3.981257E+01	3.280254E+01	6.069225E+01	2.288400E+01
F9	3.399866E+01	8.787487E+01	6.345611E+01	1.282380E+03	2.778143E+02	1.315383E+02	2.905574E+02	**8.952800E**−**02**
F10	7.488411E+02	**7.010806E**+**02**	8.291345E+02	1.664528E+03	1.249577E+03	1.130559E+03	1.208578E+03	7.258400E+02
F11	2.260349E+01	**1.612064E**+**01**	2.187918E+02	4.617377E+02	1.064103E+04	3.060763E+03	3.782708E+01	2.593500E+01
F12	2.298911E+05	7.829182E+04	8.754664E+05	1.352892E+08	1.251907E+07	4.030087E+06	4.237088E+04	**2.416100E**+**04**
F13	3.019548E+04	3.032487E+04	3.714903E+04	5.040389E+05	7.931479E+04	7.258885E+05	3.133506E+04	**2.780800E**+**04**
F14	2.559073E+04	2.240602E+04	9.555968E+03	**5.683712E**+**02**	2.514045E+04	2.288734E+04	2.687459E+04	2.884000E+03
F15	2.944716E+04	2.689140E+04	8.180000E+03	4.942168E+03	3.056713E+04	2.109167E+04	2.504587E+04	**2.831000E**+**03**
F16	3.364097E+02	3.674062E+02	3.025834E+02	4.146982E+02	6.670600E+02	4.233521E+02	6.563134E+02	**2.706500E**+**02**
F17	5.954910E+01	6.051650E+01	5.279214E+01	1.753425E+02	3.483908E+02	1.360671E+02	3.679061E+02	**3.812400E**+**01**
F18	3.684615E+04	3.832474E+04	**2.013971E**+**04**	1.676919E+06	4.657002E+04	2.755740E+06	4.043231E+04	3.788600E+04
F19	2.915792E+04	2.473200E+04	1.718581E+04	**1.279821E**+**04**	3.102943E+04	3.946864E+05	3.067541E+04	2.228400E+04
F20	**1.416370E**+**01**	1.719463E+01	1.849811E+01	1.774211E+02	4.013346E+01	5.798615E+01	3.715748E+01	3.247600E+01
F21	2.483210E+02	2.386874E+02	2.282644E+02	**1.719495E**+**02**	2.858592E+02	2.513394E+02	2.880414E+02	2.228400E+02
F22	1.213739E+03	1.055891E+03	3.270883E+02	3.557524E+02	1.562200E+03	2.136217E+02	1.767806E+03	**1.069600E**+**02**
F23	**3.390334E**+**02**	3.407041E+02	3.430707E+02	4.087868E+02	5.011995E+02	3.675843E+02	4.410332E+02	3.433900E+02
F24	3.958463E+02	3.959774E+02	3.869858E+02	3.978114E+02	4.547225E+02	**3.807829E**+**02**	4.802603E+02	3.864000E+02
F25	4.627963E+02	**4.523823E**+**02**	4.548601E+02	6.287569E+02	5.360459E+02	5.044821E+02	4.723905E+02	4.953100E+02
F26	1.713723E+03	1.479190E+03	9.804514E+02	8.865611E+02	1.913963E+03	1.503976E+03	1.998004E+03	**5.398000E**+**02**
F27	4.793855E+02	4.485870E+02	**4.148601E**+**02**	4.598156E+02	5.078251E+02	4.249553E+02	5.162868E+02	4.275500E+02
F28	6.118218E+02	9.318129E+02	**5.425620E**+**02**	6.939276E+02	6.467194E+02	7.525179E+02	9.361800E+02	6.464800E+02
F29	3.651053E+02	**3.199481E**+**02**	4.285620E+02	4.454807E+02	5.001588E+02	3.944092E+02	5.826938E+02	3.907800E+02
F30	1.664787E+06	**9.928557E**+**05**	1.525941E+06	6.206628E+06	5.490934E+06	2.090498E+06	1.437498E+06	1.131800E+06
+\−\≈	20\10\0	18\12\0	20\10\0	27\3\0	30\0\0	28\2\0	28\2\0	

Significant values are in [bold].

**Table 4:** The comparison results of the algorithms with D = 10 on the mean value.

	HS	IHS	GHS	SGHS	NGHS	IGHS	IMGHS	NIGHS
F1	3.082666E+03	**2.020484E**+**03**	2.478741E+06	1.795477E+09	8.715283E+05	2.412697E+08	3.094714E+03	2.901400E+03
F2	5.804381E+00	1.759973E−04	5.740117E+04	6.005960E+08	1.799283E+06	9.581213E+06	5.267846E−03	**2.523500E**−**05**
F3	1.881519E+02	**0.000000E**+**00**	4.781256E+02	8.778123E+03	2.860037E+04	2.492921E+02	3.258088E−04	**0.000000E**+**00**
F4	7.900697E+00	1.437521E+00	2.025659E+01	1.659683E+02	3.052521E+01	4.171601E+01	8.607948E+00	**2.071000E**−**02**
F5	**1.030219E**+**01**	1.122213E+01	1.309275E+01	6.800260E+01	2.191329E+01	2.266335E+01	2.639565E+01	1.253900E+01
F6	9.403317E−02	**8.171556E**−**05**	1.009651E+00	3.474500E+01	3.716404E−01	5.500271E+00	5.574768E−01	1.836300E−04
F7	2.613325E+01	2.823423E+01	3.064339E+01	1.586002E+02	3.746180E+01	3.268580E+01	4.071237E+01	**2.577100E**+**01**
F8	1.192863E+01	**1.173140E**+**01**	1.304089E+01	6.557195E+01	2.240011E+01	1.539718E+01	2.897081E+01	1.212100E+01
F9	7.300692E+00	3.166298E+00	9.094351E+00	8.211847E+02	3.929799E+01	5.198562E+01	8.327323E+01	**1.755500E**−**03**
F10	**2.944702E**+**02**	3.322621E+02	3.773298E+02	1.396437E+03	6.334111E+02	4.719387E+02	7.477017E+02	3.995800E+02
F11	8.778824E+00	**7.199975E**+**00**	5.350349E+01	2.732072E+02	1.181143E+03	3.139861E+02	1.560647E+01	8.246600E+00
F12	2.944940E+04	1.821888E+04	1.674412E+05	4.448335E+07	2.827314E+06	1.087194E+06	1.410872E+04	**9.033500E**+**03**
F13	1.127389E+04	9.387997E+03	1.056504E+04	1.098660E+05	1.227980E+04	3.917592E+04	1.104157E+04	**6.631300E**+**03**
F14	4.350979E+03	3.530541E+03	1.125284E+03	**2.470030E**+**02**	7.603385E+03	3.685508E+03	7.249377E+03	6.237800E+02
F15	7.059733E+03	3.964496E+03	1.788507E+03	2.249620E+03	7.506221E+03	5.939953E+03	6.494466E+03	**4.739500E**+**02**
F16	1.055355E+02	1.103884E+02	1.250154E+02	2.347308E+02	3.097281E+02	1.762647E+02	2.802027E+02	**5.922600E**+**01**
F17	1.426723E+01	**1.083553E**+**01**	1.634955E+01	1.195494E+02	7.421131E+01	3.055719E+01	7.351753E+01	1.744200E+01
F18	1.005348E+04	1.008759E+04	**6.560328E**+**03**	2.954954E+05	1.202098E+04	6.120147E+04	1.539021E+04	1.615400E+04
F19	6.979819E+03	4.296880E+03	3.762027E+03	3.439519E+03	1.043762E+04	1.699884E+04	7.224720E+03	**3.131800E**+**03**
F20	**5.141061E**+**00**	5.640003E+00	9.546239E+00	1.241029E+02	1.569869E+01	1.933959E+01	1.529432E+01	9.287800E+00
F21	2.159700E+02	2.157789E+02	1.602912E+02	**1.408387E**+**02**	2.304008E+02	1.764384E+02	2.313063E+02	1.695400E+02
F22	2.175381E+02	2.539634E+02	1.075333E+02	2.248813E+02	4.642687E+02	1.259881E+02	5.056432E+02	**1.028900E**+**02**
F23	3.202880E+02	3.191764E+02	3.209541E+02	3.684810E+02	3.371499E+02	3.269583E+02	3.472802E+02	**3.180100E**+**02**
F24	3.428243E+02	3.461528E+02	3.318026E+02	**2.917620E**+**02**	3.652043E+02	3.377379E+02	3.505659E+02	3.442900E+02
F25	4.379333E+02	4.344448E+02	4.347495E+02	5.395597E+02	**4.284174E**+**02**	4.565985E+02	4.304581E+02	4.363700E+02
F26	6.279758E+02	7.498397E+02	3.978881E+02	7.169434E+02	9.600913E+02	4.807348E+02	9.108461E+02	**3.257400E**+**02**
F27	4.008272E+02	4.022733E+02	**3.994426E**+**02**	4.419296E+02	4.369575E+02	4.058366E+02	4.316295E+02	4.033500E+02
F28	4.776499E+02	4.413097E+02	**4.266373E**+**02**	5.770697E+02	5.160939E+02	4.758310E+02	4.808126E+02	5.333500E+02
F29	2.736831E+02	**2.717692E**+**02**	2.849967E+02	3.953824E+02	3.862390E+02	2.931999E+02	3.607184E+02	2.823300E+02
F30	3.606427E+05	**6.365299E**+**04**	4.365094E+05	3.130071E+06	1.119669E+06	4.102011E+05	2.741200E+05	1.015300E+05
+\−\≈	20\7\3	15\8\7	22\8\0	27\3\0	27\3\0	28\2\0	27\3\0	

Significant values are in [bold].

**Table 5:** The comparison results of the algorithms with D = 10 on the standard deviation (SD).

	HS	IHS	GHS	SGHS	NGHS	IGHS	IMGHS	NIGHS
F1	3.157117E+03	**2.238299E**+**03**	2.023615E+06	8.226284E+08	9.618513E+05	2.075218E+08	3.026722E+03	2.815500E+03
F2	3.681245E+01	1.541466E−04	1.705384E+05	6.390310E+08	7.046274E+06	4.219783E+07	5.069793E−03	**1.654800E**−**05**
F3	2.402654E+02	**0.000000E**+**00**	3.256762E+02	2.429162E+03	1.423853E+04	2.115337E+02	6.119157E−04	1.410700E−08
F4	1.511192E+01	8.287461E−01	3.109844E+01	5.539683E+01	3.714308E+01	3.495260E+01	2.167453E+01	**9.880100E**−**03**
F5	**3.868193E**+**00**	4.556921E+00	4.995662E+00	8.075138E+00	9.671734E+00	9.125628E+00	9.679946E+00	5.512600E+00
F6	5.632713E−02	**2.336301E**−**05**	3.942273E−01	5.861767E+00	3.726448E−01	2.402125E+00	6.050812E−01	1.332700E−04
F7	7.307297E+00	**5.993279E**+**00**	7.060689E+00	2.652164E+01	9.545209E+00	1.182618E+01	1.068409E+01	7.210700E+00
F8	4.333300E+00	**4.281194E**+**00**	4.307906E+00	6.531243E+00	7.631085E+00	6.254299E+00	9.852846E+00	4.723000E+00
F9	8.245834E+00	1.250318E+01	1.196078E+01	1.786334E+02	5.029972E+01	2.953663E+01	8.199820E+01	**1.253600E**−**02**
F10	**1.441302E**+**02**	1.589762E+02	1.745732E+02	1.754113E+02	2.382769E+02	2.236239E+02	2.231638E+02	1.849900E+02
F11	4.896088E+00	**3.790836E**+**00**	5.305782E+01	8.207131E+01	2.104694E+03	6.062726E+02	9.259823E+00	4.643800E+00
F12	3.841892E+04	1.698214E+04	1.574112E+05	2.703376E+07	3.172433E+06	1.148226E+06	1.054213E+04	**5.069700E**+**03**
F13	1.001119E+04	1.012826E+04	1.145075E+04	1.074968E+05	1.402090E+04	1.256247E+05	1.037862E+04	**6.992600E**+**03**
F14	7.006648E+03	5.522954E+03	2.147176E+03	**1.221350E**+**02**	7.554092E+03	5.646284E+03	7.759361E+03	6.328100E+02
F15	8.471770E+03	5.883461E+03	1.836074E+03	1.172249E+03	8.506765E+03	5.941242E+03	7.767935E+03	**6.915500E**+**02**
F16	9.658785E+01	1.052367E+02	9.207001E+01	7.649027E+01	1.624273E+02	1.167408E+02	1.582717E+02	**7.375800E**+**01**
F17	1.372130E+01	1.547942E+01	1.236455E+01	2.700804E+01	7.083862E+01	2.538505E+01	7.767468E+01	**7.678400E**+**00**
F18	8.607798E+03	8.795645E+03	**5.661971E**+**03**	3.162489E+05	1.154060E+04	3.849966E+05	1.098832E+04	9.371700E+03
F19	9.031860E+03	5.987647E+03	4.129975E+03	**2.852037E**+**03**	9.054784E+03	5.471260E+04	8.560898E+03	4.317100E+03
F20	5.931667E+00	6.098234E+00	**4.069779E**+**00**	2.536000E+01	1.125335E+01	1.157770E+01	1.088507E+01	8.020000E+00
F21	2.463861E+01	2.474735E+01	3.462078E+01	**1.387511E**+**01**	3.935563E+01	5.749930E+01	4.172461E+01	5.640000E+01
F22	2.726847E+02	2.993853E+02	4.118625E+01	6.374358E+01	5.246328E+02	2.484963E+01	5.577688E+02	**1.696500E**+**00**
F23	7.871882E+00	7.423315E+00	**6.685624E**+**00**	4.039476E+01	2.756917E+01	1.347747E+01	2.577728E+01	8.080700E+00
F24	6.260154E+01	5.623161E+01	5.295424E+01	4.201941E+01	9.080895E+01	6.163622E+01	1.017737E+02	**3.656500E**+**01**
F25	2.068780E+01	2.085310E+01	3.109816E+01	4.207752E+01	5.342638E+01	**1.519722E**+**01**	4.447559E+01	2.354700E+01
F26	5.016889E+02	5.039721E+02	1.535856E+02	**9.299323E**+**01**	6.095904E+02	2.173363E+02	5.849425E+02	1.306300E+02
F27	1.552200E+01	1.267574E+01	**5.787664E**+**00**	9.950950E+00	3.419072E+01	6.548021E+00	2.664334E+01	8.745300E+00
F28	1.175870E+02	1.416208E+02	**4.324284E**+**01**	5.978654E+01	1.247084E+02	1.184220E+02	1.602807E+02	1.254100E+02
F29	**2.292976E**+**01**	2.362148E+01	3.529465E+01	3.102604E+01	6.206546E+01	3.106894E+01	7.814361E+01	3.202800E+01
F30	4.625975E+05	**1.919057E**+**05**	3.845446E+05	1.477886E+06	1.116548E+06	4.204463E+05	4.634590E+05	2.947200E+05
+\−\≈	20\7\3	15\11\4	19\6\5	23\6\1	28\2\0	26\1\3	29\1\0	

Significant values are in [bold].

**Table 6:** The comparison results of the algorithms with D = 30 on the best value.

	HS	IHS	GHS	SGHS	NGHS	IGHS	IMGHS	NIGHS
F1	1.068523E+02	**2.046065E**−**01**	9.027079E+07	2.545320E+10	3.668090E+04	2.327563E+09	2.212518E+02	2.552100E+00
F2	3.034429E+07	4.843791E+01	3.866970E+17	4.302386E+35	3.243556E+08	7.444072E+24	**0.000000E**+**00**	7.144800E−06
F3	2.580146E+03	1.622417E+03	1.989163E+04	6.270746E+04	4.981456E+04	1.023267E+04	1.356627E−03	**3.381400E**−**06**
F4	4.362691E−01	4.000278E+00	1.012565E+02	3.075531E+03	1.140058E+00	4.984801E+02	5.688817E−03	**2.648200E**−**03**
F5	3.581881E+01	4.278316E+01	6.074284E+01	3.483356E+02	7.273690E+01	2.208777E+02	8.954596E+01	**3.382900E**+**01**
F6	1.296731E−01	**7.662107E**−**04**	3.492141E+00	6.483407E+01	3.378435E−03	2.068939E+01	1.156592E−02	3.784700E−03
F7	8.010618E+01	9.042612E+01	1.638883E+02	8.607841E+02	7.930792E+01	3.158510E+02	1.133163E+02	**5.795600E**+**01**
F8	4.576810E+01	4.377812E+01	8.695433E+01	3.197685E+02	7.369765E+01	1.866902E+02	8.357645E+01	**2.686400E**+**01**
F9	3.353991E+01	4.456849E+01	1.934407E+02	8.462429E+03	2.741974E+02	1.793276E+03	2.451158E+02	**2.490100E**+**01**
F10	1.236777E+03	**1.087651E**+**03**	2.579004E+03	6.482077E+03	1.692920E+03	6.208016E+03	1.777147E+03	1.316400E+03
F11	2.196301E+01	1.702465E+01	2.052165E+02	3.259494E+03	1.602049E+02	1.811461E+02	2.092876E+01	**1.633100E**+**01**
F12	8.593028E+04	1.016238E+05	2.527995E+06	9.455021E+08	3.712921E+05	3.290853E+07	**3.400053E**+**03**	4.489000E+04
F13	2.157118E+02	**1.104996E**+**02**	5.972810E+04	2.433333E+08	2.612297E+04	1.109344E+05	8.691712E+02	2.464800E+02
F14	8.903657E+02	**3.633828E**+**02**	1.016913E+04	1.280052E+05	1.025964E+05	1.030058E+04	1.607166E+03	1.441700E+03
F15	6.008367E+01	**4.002869E**+**01**	3.699453E+03	2.593205E+07	4.898971E+03	2.477443E+03	2.816662E+02	2.736200E+02
F16	3.267963E+02	4.331046E+02	4.928217E+02	2.018867E+03	5.285891E+02	1.056037E+03	6.953776E+02	**2.480000E**+**02**
F17	4.981539E+01	6.811743E+01	9.120146E+01	8.697274E+02	1.681502E+02	1.278035E+02	1.976489E+02	**3.839900E**+**01**
F18	**7.365750E**+**03**	1.806577E+04	6.873488E+04	1.712025E+06	8.417784E+04	7.097923E+04	1.182169E+04	1.315000E+04
F19	2.007914E+01	**1.495560E**+**01**	8.764441E+03	7.474144E+07	1.433116E+03	8.792379E+03	4.596323E+01	6.174900E+02
F20	1.652455E+02	**4.352280E**+**01**	1.932236E+02	5.804556E+02	1.950264E+02	9.454303E+01	1.960127E+02	7.586100E+01
F21	2.431781E+02	2.530248E+02	2.725541E+02	5.161222E+02	2.703084E+02	3.943160E+02	2.723433E+02	**2.286200E**+**02**
F22	1.000269E+02	1.000010E+02	1.623281E+02	3.500901E+03	1.012834E+02	6.386560E+02	1.000343E+02	**1.000000E**+**02**
F23	3.911640E+02	3.920824E+02	4.311308E+02	7.595799E+02	4.131467E+02	5.676154E+02	4.082974E+02	**3.819800E**+**02**
F24	4.613792E+02	4.640112E+02	5.274326E+02	8.631629E+02	5.889263E+02	6.615161E+02	5.684989E+02	**4.591900E**+**02**
F25	3.838279E+02	3.834947E+02	4.032079E+02	1.166790E+03	3.844018E+02	5.490780E+02	3.839042E+02	**3.834000E**+**02**
F26	3.000524E+02	3.000018E+02	6.298026E+02	5.952464E+03	2.058838E+02	1.551585E+03	2.002229E+02	**2.000200E**+**02**
F27	**4.946378E**+**02**	5.067482E+02	5.229982E+02	7.443608E+02	5.087726E+02	5.471227E+02	5.237302E+02	5.011100E+02
F28	3.999236E+02	3.909026E+02	4.634589E+02	1.777792E+03	3.989388E+02	6.168254E+02	3.000053E+02	**3.000000E**++**02**
F29	3.918434E+02	**3.793398E**+**02**	5.220060E+02	2.232862E+03	5.702581E+02	7.995088E+02	5.685322E+02	4.598300E+02
F30	3.601612E+03	**3.013336E**+**03**	5.759281E+04	2.182670E+07	6.112615E+03	2.678563E+05	3.252771E+03	3.374800E+03
+\−\≈	22\8\0	20\10\0	30\0\0	30\0\0	29\1\0	30\0\0	25\5\0	

Significant values are in [bold].

**Table 7:** The comparison results of the algorithms with D = 30 on the worst value.

	HS	IHS	GHS	SGHS	NGHS	IGHS	IMGHS	NIGHS
F1	2.072044E+04	**1.744687E**+**04**	4.643647E+08	5.800895E+10	6.277895E+05	8.835646E+09	2.096193E+04	2.081100E+04
F2	6.491563E+13	1.387694E+12	1.171382E+26	8.175159E+40	5.221311E+22	9.426971E+31	8.500180E−03	**1.288300E**−**04**
F3	1.097459E+04	1.583807E+04	4.796008E+04	1.313875E+05	2.322391E+05	3.936810E+04	9.086220E+03	**1.064900E**−**05**
F4	1.845267E+02	1.472882E+02	2.499712E+02	1.246151E+04	1.984702E+02	1.426510E+03	**6.411747E**+**01**	1.092400E+02
F5	1.213845E+02	**1.074553E**+**02**	1.502890E+02	4.622845E+02	2.201519E+02	2.973286E+02	2.318238E+02	1.333200E+02
F6	6.028584E−01	**2.172649E**−**01**	8.496970E+00	9.372626E+01	2.989244E−01	4.675106E+01	6.297507E−01	6.227700E−01
F7	**1.752455E**+**02**	1.824142E+02	2.791493E+02	1.671383E+03	2.532590E+02	4.331519E+02	3.223059E+02	1.759500E+02
F8	1.223795E+02	1.164097E+02	1.600059E+02	4.231056E+02	2.100051E+02	2.645997E+02	2.198843E+02	**9.651100E**+**01**
F9	1.587352E+03	2.042237E+03	3.370909E+03	1.650976E+04	8.091164E+03	6.002449E+03	7.129785E+03	**1.024000E**+**03**
F10	**3.143604E**+**03**	3.418866E+03	4.915291E+03	7.838171E+03	4.391321E+03	7.544039E+03	4.365324E+03	7.664400E+03
F11	5.820558E+02	**1.172088E**+**02**	1.033293E+03	8.029528E+03	1.913853E+04	1.244433E+03	1.823572E+02	1.659000E+02
F12	8.200471E+06	4.805210E+06	2.535490E+07	7.619030E+09	1.381693E+07	2.580293E+08	**1.793248E**+**05**	1.735000E+06
F13	6.110421E+04	6.116948E+04	5.716129E+06	3.828686E+09	5.436535E+05	3.431695E+06	6.214898E+04	**6.109100E**+**04**
F14	3.064803E+05	6.889374E+04	1.215528E+06	9.451569E+05	8.315814E+06	1.079273E+06	6.200836E+04	**5.158200E**+**04**
F15	4.135316E+04	3.795285E+04	8.669585E+04	4.589281E+08	2.302278E+05	2.271913E+06	3.393528E+04	**8.366400E**+**03**
F16	1.435014E+03	1.435238E+03	1.560175E+03	3.196043E+03	1.990081E+03	2.250118E+03	2.185376E+03	**1.240500E**+**03**
F17	7.576402E+02	7.571216E+02	9.397974E+02	1.670400E+03	1.101852E+03	6.877205E+02	1.412618E+03	**6.540300E**+**02**
F18	9.011841E+05	1.277854E+06	6.149307E+06	1.597349E+07	1.902476E+07	5.001350E+06	4.178598E+05	**4.082400E**+**05**
F19	5.135418E+04	**4.826647E**+**04**	1.659111E+05	5.181274E+08	1.495130E+05	1.215384E+06	5.292185E+04	5.439200E+04
F20	8.560076E+02	7.366954E+02	8.170349E+02	9.586575E+02	1.220214E+03	6.728616E+02	1.223491E+03	**5.353900E**+**02**
F21	3.681478E+02	3.525013E+02	3.868032E+02	6.211403E+02	4.084442E+02	4.787967E+02	4.814684E+02	**2.930600E**+**02**
F22	3.818090E+03	3.428020E+03	5.056001E+03	6.616301E+03	4.901014E+03	7.340415E+03	5.486943E+03	**1.034000E**+**02**
F23	4.858749E+02	4.940197E+02	5.406275E+02	1.031957E+03	6.090156E+02	6.953638E+02	6.277485E+02	**4.412300E**+**02**
F24	5.831273E+02	6.044994E+02	6.634224E+02	1.089566E+03	9.512382E+02	7.458484E+02	1.080535E+03	**5.429400E**+**02**
F25	4.521908E+02	4.270659E+02	6.406562E+02	5.158935E+03	4.706405E+02	8.113083E+02	4.497501E+02	**4.241500E**+**02**
F26	2.552411E+03	2.805988E+03	3.191595E+03	7.671484E+03	4.702895E+03	4.553354E+03	4.560004E+03	**2.125900E**+**03**
F27	5.661699E+02	5.602909E+02	5.723595E+02	1.237746E+03	6.962743E+02	6.835318E+02	6.346156E+02	**5.397700E**+**02**
F28	5.322946E+02	4.724356E+02	6.685127E+02	4.208768E+03	5.056967E+02	1.164292E+03	**4.666402E**+**02**	4.832900E+02
F29	1.156735E+03	1.285325E+03	1.354054E+03	3.108322E+03	1.496460E+03	1.948742E+03	1.620953E+03	**9.737000E**+**02**
F30	2.362344E+04	7.782387E+04	3.191253E+06	4.248251E+08	1.194065E+06	3.074036E+07	2.195591E+04	**2.154600E**+**04**
+\−\≈	24\6\0	23\7\0	29\1\0	30\0\0	28\2\0	29\1\0	26\4\0	

Significant values are in [bold].

**Table 8:** The comparison results of the algorithms with D = 30 on the mean.

	HS	IHS	GHS	SGHS	NGHS	IGHS	IMGHS	NIGHS
F1	6.512962E+03	**3.788448E**+**03**	2.201741E+08	4.187384E+10	2.155905E+05	5.659333E+09	6.600335E+03	5.606100E+03
F2	6.313006E+12	1.272550E+11	2.348883E+24	4.383949E+39	1.023786E+21	2.870979E+30	1.520037E−03	**3.795500E**−**05**
F3	5.958059E+03	5.559183E+03	3.153312E+04	1.086114E+05	1.264039E+05	2.873143E+04	7.074914E+02	**5.819200E**−**06**
F4	9.644815E+01	9.164199E+01	1.778178E+02	7.875975E+03	1.134628E+02	8.485300E+02	**2.162090E**+**01**	7.072300E+01
F5	**6.788279E**+**01**	7.146847E+01	1.212779E+02	4.160562E+02	1.245538E+02	2.625764E+02	1.518558E+02	6.818900E+01
F6	3.113504E−01	**2.940543E**−**02**	5.582615E+00	8.134738E+01	9.284275E−02	3.146261E+01	1.508032E−01	7.472000E−02
F7	1.206463E+02	1.341869E+02	2.201829E+02	1.318888E+03	1.546895E+02	3.605476E+02	2.085803E+02	**1.117400E**+**02**
F8	7.051185E+01	7.467204E+01	1.213927E+02	3.830139E+02	1.344235E+02	2.383102E+02	1.482902E+02	**6.588400E**+**01**
F9	3.690116E+02	4.874836E+02	9.324867E+02	1.346845E+04	2.269432E+03	3.303545E+03	3.141242E+03	**2.938200E**+**02**
F10	**2.126315E**+**03**	2.187192E+03	3.875644E+03	7.220065E+03	2.973548E+03	6.888711E+03	3.180011E+03	3.077200E+03
F11	1.115466E+02	**5.442799E**+**01**	4.641186E+02	5.954367E+03	4.217659E+03	4.149224E+02	9.620332E+01	7.202700E+01
F12	2.661634E+06	1.888724E+06	9.160858E+06	4.185204E+09	5.814084E+06	9.975334E+07	**4.821182E**+**04**	7.544200E+05
F13	2.254860E+04	**1.638573E**+**04**	5.325643E+05	1.822527E+09	1.723152E+05	9.455015E+05	2.234773E+04	3.627800E+04
F14	5.979258E+04	2.266145E+04	3.860673E+05	4.539325E+05	2.103714E+06	2.091559E+05	2.485078E+04	**2.107400E**+**04**
F15	8.406822E+03	1.046976E+04	3.401620E+04	1.749924E+08	5.761334E+04	1.326671E+05	7.526183E+03	**4.247100E**+**03**
F16	9.812774E+02	9.293102E+02	1.073805E+03	2.823986E+03	1.317158E+03	1.831384E+03	1.377878E+03	**7.584800E**+**02**
F17	3.933099E+02	4.375711E+02	4.936058E+02	1.311008E+03	6.827482E+02	3.164039E+02	7.040197E+02	**2.767900E**+**02**
F18	2.232848E+05	2.136390E+05	1.199700E+06	8.745486E+06	2.969587E+06	1.104413E+06	1.338393E+05	**9.344600E**+**04**
F19	1.181221E+04	1.245201E+04	6.062635E+04	2.417767E+08	2.630492E+04	1.870948E+05	9.783248E+03	**3.821300E**+**03**
F20	4.700964E+02	4.338644E+02	4.858089E+02	8.112184E+02	6.592286E+02	3.414967E+02	6.985217E+02	**2.327700E**+**02**
F21	2.782765E+02	2.850319E+02	3.172070E+02	5.792569E+02	3.382485E+02	4.437873E+02	3.518632E+02	**2.557100E**+**02**
F22	2.201432E+03	2.262349E+03	3.112827E+03	5.493993E+03	3.431655E+03	1.355800E+03	3.499604E+03	**1.002100E**+**02**
F23	4.244062E+02	4.372907E+02	4.824612E+02	9.291052E+02	4.924876E+02	6.343143E+02	5.096321E+02	**4.124100E**+**02**
F24	5.150083E+02	5.150276E+02	5.810737E+02	1.006163E+03	7.328469E+02	7.016840E+02	7.786153E+02	**4.974700E**+**02**
F25	3.947690E+02	3.912484E+02	4.654848E+02	3.380473E+03	4.055069E+02	6.429080E+02	4.001704E+02	**3.868300E**+**02**
F26	1.900689E+03	1.925130E+03	2.378049E+03	6.954850E+03	2.445974E+03	3.519399E+03	2.809112E+03	**1.498000E**+**03**
F27	5.258889E+02	5.270481E+02	5.453750E+02	1.049146E+03	5.575036E+02	6.046771E+02	5.717067E+02	**5.190100E**+**02**
F28	4.349334E+02	4.240114E+02	5.355993E+02	3.270455E+03	4.363745E+02	7.654562E+02	**3.480443E**+**02**	3.483500E+02
F29	8.140057E+02	8.000748E+02	9.379534E+02	2.634272E+03	1.027492E+03	1.316252E+03	1.035109E+03	**6.808000E**+**02**
F30	1.060983E+04	1.041189E+04	6.062064E+05	1.998033E+08	4.819860E+04	2.802429E+06	9.040434E+03	8.199600E+03
+\−\≈	27\3\0	25\5\0	30\0\0	30\0\0	29\1\0	30\0\0	26\4\0	

Significant values are in [bold].

**Table 9:** The comparison results of the algorithms with D = 30 on the standard deviation(SD).

	HS	IHS	GHS	SGHS	NGHS	IGHS	IMGHS	NIGHS
F1	6.462226E+03	**4.411202E**+**03**	7.985117E+07	9.242748E+09	1.432383E+05	1.254290E+09	6.430167E+03	6.551700E+03
F2	1.422517E+13	3.070156E+11	1.639664E+25	1.222324E+40	7.311298E+21	1.319634E+31	2.245412E−03	**2.157000E**−**05**
F3	2.247776E+03	2.758061E+03	6.615690E+03	1.356305E+04	4.575386E+04	5.469181E+03	1.533124E+03	**1.890800E**−**06**
F4	3.599719E+01	2.880974E+01	3.519982E+01	2.277040E+03	4.169752E+01	1.792991E+02	**2.595811E**+**01**	2.829300E+01
F5	1.975193E+01	**1.593070E**+**01**	2.027662E+01	2.590202E+01	3.307842E+01	1.706140E+01	3.870637E+01	1.989700E+01
F6	9.760512E−02	**4.010523E**−**02**	1.126594E+00	6.662674E+00	7.786286E−02	4.933538E+00	1.096524E−01	1.031300E−01
F7	**2.227391E**+**01**	2.292746E+01	2.885607E+01	1.890464E+02	3.122018E+01	2.663446E+01	4.599143E+01	2.494800E+01
F8	1.864943E+01	1.733408E+01	2.068358E+01	1.946118E+01	3.515333E+01	**1.479549E**+**01**	3.326178E+01	1.762000E+01
F9	3.144256E+02	3.763686E+02	5.464909E+02	1.784927E+03	1.708922E+03	9.858191E+02	1.500953E+03	**2.140900E**+**02**
F10	4.539781E+02	5.326363E+02	5.192709E+02	**2.762411E**+**02**	5.247567E+02	3.037175E+02	5.213756E+02	1.581000E+03
F11	9.185669E+01	**2.956277E**+**01**	1.902684E+02	1.045637E+03	4.234318E+03	1.952812E+02	3.776199E+01	3.529000E+01
F12	2.104029E+06	1.226197E+06	5.041376E+06	1.521853E+09	3.704490E+06	4.609968E+07	**3.821718E**+**04**	4.885500E+05
F13	2.132337E+04	**1.971646E**+**04**	1.099157E+06	8.939013E+08	1.326505E+05	7.684824E+05	1.990755E+04	2.212600E+04
F14	6.268087E+04	1.813784E+04	2.787775E+05	2.169405E+05	2.010037E+06	2.326886E+05	1.665911E+04	**1.105000E**+**04**
F15	1.068804E+04	1.105467E+04	1.984252E+04	9.026478E+07	5.132744E+04	3.480844E+05	8.681166E+03	**1.723900E**+**03**
F16	2.855997E+02	2.743891E+02	2.867361E+02	2.233444E+02	3.890566E+02	2.423724E+02	3.116741E+02	**2.195200E**+**02**
F17	1.691612E+02	1.743036E+02	1.796955E+02	1.796218E+02	2.144988E+02	**1.496326E**+**02**	2.723753E+02	1.703700E+02
F18	2.148476E+05	2.497883E+05	1.198498E+06	3.727518E+06	3.867678E+06	1.032170E+06	9.161686E+04	**7.762200E**+**04**
F19	1.369357E+04	1.361182E+04	3.887535E+04	1.185807E+08	2.494422E+04	2.443071E+05	1.214816E+04	**7.315400E**+**03**
F20	1.707209E+02	1.562894E+02	1.389187E+02	**8.743837E**+**01**	2.466869E+02	1.337035E+02	2.151317E+02	9.825400E+01
F21	2.002380E+01	2.221640E+01	2.324984E+01	2.413891E+01	3.424504E+01	1.816649E+01	3.911371E+01	**1.466600E**+**01**
F22	1.100165E+03	1.089402E+03	1.784682E+03	6.252949E+02	1.234092E+03	1.725858E+03	1.248683E+03	**7.391200E**−**01**
F23	1.859161E+01	2.215060E+01	2.115037E+01	5.308913E+01	4.127987E+01	2.340409E+01	4.218732E+01	**1.359800E**+**01**
F24	2.642286E+01	2.775383E+01	3.792034E+01	5.741005E+01	8.814387E+01	1.973049E+01	1.248930E+02	**1.732800E**+**01**
F25	1.487534E+01	1.088684E+01	4.283816E+01	8.915368E+02	1.991330E+01	5.869375E+01	1.806065E+01	**5.654300E**+**00**
F26	**3.487429E**+**02**	5.369188E+02	5.224857E+02	4.368112E+02	1.225353E+03	9.034928E+02	1.190094E+03	5.527300E+02
F27	1.351662E+01	1.094871E+01	1.183669E+01	1.096920E+02	3.211690E+01	3.028793E+01	2.820783E+01	**9.259300E**+**00**
F28	2.838074E+01	**1.846651E**+**01**	4.350773E+01	5.851014E+02	2.520755E+01	9.460924E+01	6.148169E+01	6.570600E+01
F29	1.911047E+02	2.180168E+02	2.141639E+02	2.039071E+02	2.394294E+02	2.685951E+02	2.440876E+02	**1.603000E**+**02**
F30	4.930295E+03	1.069675E+04	6.252874E+05	1.049200E+08	1.644382E+05	4.453103E+06	4.511316E+03	**4.406700E**+**03**
+\−\≈	20\7\3	20\8\2	27\3\0	27\3\0	27\3\0	26\4\0	24\5\1	

Significant values are in [bold].

**Table 10:** The comparison results of the algorithms with D = 50 on the best value.

	HS	IHS	GHS	SGHS	NGHS	IGHS	IMGHS	NIGHS
F1	5.666325E+06	1.525691E+01	2.651446E+09	6.463797E+10	9.865221E+04	2.478963E+10	2.695192E+02	**1.243900E**+**01**
F2	6.304529E+23	1.640737E+23	6.558419E+44	8.544548E+67	2.590667E+11	2.530122E+51	**9.038759E**−**06**	3.278400E−05
F3	1.277708E+04	1.274871E+04	7.540114E+04	1.459917E+05	6.543419E+04	5.861526E+04	7.493945E−04	**1.370900E**−**04**
F4	1.213797E+02	9.645934E+01	4.663119E+02	1.719059E+04	3.325200E+01	3.010765E+03	**1.693984E**−**03**	6.645500E+00
F5	**9.042231E**+**01**	9.466620E+01	2.515225E+02	6.846155E+02	1.344391E+02	4.817152E+02	1.711324E+02	9.352600E+01
F6	5.247919E−01	5.823522E−01	1.156537E+01	7.856944E+01	**1.169702E**−**03**	4.687130E+01	3.270827E−02	4.729100E−02
F7	1.722506E+02	1.790776E+02	4.892317E+02	2.163647E+03	1.759570E+02	7.233945E+02	2.672404E+02	**1.536400E**+**02**
F8	9.911058E+01	8.559724E+01	2.765768E+02	6.682898E+02	1.473926E+02	4.708489E+02	1.482484E+02	**6.964700E**+**01**
F9	5.435234E+02	**2.787040E**+**02**	2.222393E+03	3.691836E+04	1.088541E+03	1.216300E+04	2.109018E+03	5.704900E+02
F10	2.783521E+03	**2.524240E**+**03**	7.945176E+03	1.223400E+04	3.933811E+03	1.216510E+04	4.037374E+03	2.970600E+03
F11	9.157539E+01	**5.765687E**+**01**	5.888955E+02	1.159133E+04	1.709634E+02	1.231683E+03	8.089060E+01	8.160600E+01
F12	1.260697E+06	4.069601E+06	8.937306E+07	1.571582E+10	2.709029E+06	1.937340E+09	**4.187495E**+**04**	4.874600E+05
F13	5.091521E+02	3.091342E+02	3.747884E+05	2.862798E+09	3.938280E+04	3.210113E+06	**2.481001E**+**02**	5.574800E+02
F14	**1.605743E**+**03**	5.929771E+03	3.457225E+04	2.275779E+06	1.046563E+06	1.558671E+05	3.355307E+03	7.671800E+03
F15	1.573028E+02	**9.659128E**+**01**	2.640743E+04	9.147456E+08	1.439264E+04	4.145002E+04	2.215135E+02	3.024200E+03
F16	8.165562E+02	**6.379410E**+**02**	1.111169E+03	3.736912E+03	1.261271E+03	2.789208E+03	1.391734E+03	6.593500E+02
F17	4.394151E+02	**3.876294E**+**02**	7.616631E+02	3.801946E+03	8.354612E+02	1.519869E+03	8.734917E+02	6.437500E+02
F18	1.566773E+05	5.212841E+04	1.032658E+06	1.653815E+07	1.216400E+06	6.996651E+05	**3.788443E**+**04**	7.345600E+04
F19	7.703162E+01	8.424755E+01	1.445032E+04	1.409187E+08	1.222709E+03	1.578218E+04	**6.194786E**+**01**	1.898100E+03
F20	4.537277E+02	3.205586E+02	4.901425E+02	1.536136E+03	6.503819E+02	3.682803E+02	5.548137E+02	**1.989100E**+**02**
F21	2.989820E+02	**2.827565E**+**02**	4.705390E+02	9.290004E+02	3.225218E+02	6.654872E+02	3.597713E+02	2.875600E+02
F22	3.824043E+03	3.455692E+03	8.378088E+03	1.291272E+04	4.443062E+03	3.797203E+03	4.462614E+03	**1.000100E**+**02**
F23	5.398951E+02	**5.125670E**+**02**	7.426499E+02	1.442461E+03	6.022895E+02	1.002041E+03	6.169710E+02	5.236800E+02
F24	5.813216E+02	5.783206E+02	9.052604E+02	1.546477E+03	8.884984E+02	1.051181E+03	8.300935E+02	**5.680400E**+**02**
F25	5.280388E+02	5.164477E+02	8.024966E+02	7.726843E+03	5.267844E+02	2.258547E+03	**4.290447E**+**02**	4.607900E+02
F26	4.035850E+02	2.189507E+03	4.568824E+03	1.103818E+04	3.012631E+02	5.027276E+03	**3.000701E**+**02**	2.153800E+03
F27	**5.521775E**+**02**	5.741683E+02	7.269276E+02	2.003946E+03	7.107031E+02	1.140944E+03	7.306888E+02	5.992300E+02
F28	4.708653E+02	4.802526E+02	7.536427E+02	6.566306E+03	4.771654E+02	2.064586E+03	**4.533519E**+**02**	4.588500E+02
F29	**4.937402E**+**02**	5.565139E+02	7.415581E+02	4.487446E+03	8.347101E+02	2.705094E+03	7.619448E+02	5.159200E+02
F30	6.698851E+05	7.418305E+05	2.346566E+06	7.105646E+08	**6.371493E**+**05**	1.759877E+07	6.754411E+05	6.521100E+05
+\−\≈	19\11\0	17\12\1	30\0\0	30\0\0	27\3\0	30\0\0	17\9\4	

Significant values are in [bold].

**Table 11:** The comparison results of the algorithms with D = 50 on the worst value.

	HS	IHS	GHS	SGHS	NGHS	IGHS	IMGHS	NIGHS
F1	3.165234E+07	1.211186E+06	6.554941E+09	1.469362E+11	7.122064E+05	3.960310E+10	3.212907E+04	**2.348900E**+**04**
F2	8.296359E+41	1.388760E+35	9.010779E+57	5.805540E+74	6.572528E+51	3.242183E+63	**1.086282E**−**03**	1.352600E−03
F3	3.280005E+04	3.525283E+04	1.329954E+05	2.636805E+05	2.852862E+05	1.154549E+05	**8.779205E**+**03**	1.256800E+04
F4	3.544831E+02	3.769739E+02	1.076862E+03	4.074119E+04	2.484170E+02	6.475148E+03	**1.179991E**+**02**	1.804000E+02
F5	2.268882E+02	**1.986447E**+**02**	4.347801E+02	8.919588E+02	3.124606E+02	5.932317E+02	3.900188E+02	2.298300E+02
F6	1.702790E+00	2.389538E+00	1.992304E+01	1.122076E+02	**1.821650E**−**01**	6.923466E+01	2.954765E−01	3.772300E−01
F7	3.621688E+02	3.688003E+02	6.665588E+02	3.500934E+03	3.837618E+02	9.042958E+02	5.273219E+02	**3.144500E**+**02**
F8	2.278035E+02	2.063107E+02	4.047163E+02	8.799094E+02	4.418241E+02	5.728057E+02	3.860409E+02	**2.009800E**+**02**
F9	**3.837860E**+**03**	4.302979E+03	1.396394E+04	5.504242E+04	1.209624E+04	2.841323E+04	1.979137E+04	1.413200E+04
F10	**5.846698E**+**03**	5.931644E+03	1.197453E+04	1.418743E+04	6.980447E+03	1.375193E+04	6.435420E+03	1.375200E+04
F11	1.300138E+03	1.041661E+03	3.643487E+03	2.724348E+04	2.134862E+04	3.724668E+03	2.916687E+02	**2.204000E**+**02**
F12	2.422872E+07	3.867203E+07	5.269063E+08	5.436939E+10	3.080194E+07	4.968224E+09	**6.745627E**+**05**	5.295100E+06
F13	3.818491E+04	**3.457127E**+**04**	4.117037E+06	1.869300E+10	1.698476E+06	6.365039E+07	3.823958E+04	3.795900E+04
F14	6.367041E+05	1.940049E+05	6.812386E+06	1.576265E+07	1.837013E+07	3.621086E+06	**9.068309E**+**04**	2.722000E+05
F15	2.290922E+04	**1.807920E**+**04**	4.080654E+05	5.066036E+09	2.761270E+05	2.456934E+06	2.072477E+04	1.829000E+04
F16	2.749820E+03	2.411354E+03	3.164141E+03	6.142292E+03	3.169898E+03	4.185834E+03	3.130631E+03	**2.238500E**+**03**
F17	1.864170E+03	1.752337E+03	2.052643E+03	6.178939E+03	2.673709E+03	2.522315E+03	2.568920E+03	**1.565700E**+**03**
F18	8.717833E+06	4.023688E+06	3.772368E+07	7.369686E+07	4.130364E+07	1.844258E+07	**3.485325E**+**05**	9.428300E+05
F19	**4.246256E**+**04**	4.250573E+04	1.894741E+05	2.280208E+09	5.664085E+04	3.943316E+06	4.256946E+04	4.257200E+04
F20	1.594840E+03	**1.591007E**+**03**	1.834600E+03	2.222266E+03	2.149430E+03	2.045652E+03	1.933758E+03	1.959800E+03
F21	4.005323E+02	**3.977943E**+**02**	6.388313E+02	1.105549E+03	5.756700E+02	8.071128E+02	5.653965E+02	4.081800E+02
F22	**6.466861E**+**03**	6.958919E+03	1.247217E+04	1.458986E+04	7.708544E+03	1.436021E+04	8.561889E+03	1.419700E+04
F23	7.055391E+02	7.683508E+02	9.426123E+02	1.823880E+03	8.389685E+02	1.182242E+03	9.509353E+02	**6.682500E**+**02**
F24	8.829180E+02	9.220994E+02	1.038913E+03	2.069423E+03	1.708605E+03	1.238148E+03	1.649787E+03	**7.674300E**+**02**
F25	6.435229E+02	6.565677E+02	1.294107E+03	2.294790E+04	6.480245E+02	4.632676E+03	6.433201E+02	**5.809700E**+**02**
F26	**3.563530E**+**03**	3.616021E+03	6.093491E+03	1.692760E+04	5.710282E+03	8.813710E+03	7.749841E+03	3.977000E+03
F27	8.751784E+02	9.725776E+02	1.032377E+03	2.997552E+03	1.184249E+03	1.627493E+03	1.354702E+03	**8.176200E**+**02**
F28	6.625035E+02	6.535327E+02	1.458742E+03	1.201106E+04	6.180835E+02	3.615580E+03	6.379328E+02	**5.888200E**+**02**
F29	1.680448E+03	**1.602945E**+**03**	2.280341E+03	1.156650E+04	2.319713E+03	4.008847E+03	2.409903E+03	1.634700E+03
F30	2.454504E+06	2.144837E+06	1.215037E+07	3.368960E+09	3.582752E+08	1.095137E+08	1.540380E+06	**1.162500E**+**06**
+\−\≈	22\8\0	18\12\0	26\4\0	30\0\0	26\4\0	29\1\0	19\8\3	

Significant values are in [bold].

**Table 12:** The comparison results of the algorithms with D = 50 on the mean value.

	HS	IHS	GHS	SGHS	NGHS	IGHS	IMGHS	NIGHS
F1	1.590451E+07	3.907115E+04	4.805588E+09	1.155079E+11	2.699914E+05	3.196065E+10	7.451278E+03	**5.319800E**+**03**
F2	1.629235E+40	3.397521E+33	1.799400E+56	4.725988E+73	1.288731E+50	1.464518E+62	3.778952E−04	**1.251700E**−**04**
F3	2.270806E+04	2.274243E+04	1.010417E+05	2.266084E+05	1.666602E+05	9.423376E+04	**1.311104E**+**03**	2.060300E+03
F4	2.140085E+02	1.953281E+02	7.345380E+02	2.763835E+04	1.432148E+02	4.920724E+03	**3.369613E**+**01**	7.086200E+01
F5	1.433658E+02	**1.432072E**+**02**	3.390869E+02	8.261421E+02	2.266139E+02	5.332249E+02	2.632654E+02	1.542200E+02
F6	1.142637E+00	1.242440E+00	1.476749E+01	1.027234E+02	**5.351880E**−**02**	5.632131E+01	1.312583E−01	1.676800E−01
F7	2.650513E+02	2.629117E+02	5.639037E+02	2.965662E+03	2.723209E+02	7.983279E+02	3.835455E+02	**2.444300E**+**02**
F8	**1.380375E**+**02**	1.463104E+02	3.409246E+02	8.212735E+02	2.437805E+02	5.263346E+02	2.620628E+02	1.429500E+02
F9	**1.416839E**+**03**	1.753497E+03	6.316071E+03	4.626831E+04	5.332417E+03	1.822826E+04	8.448620E+03	2.105600E+03
F10	**4.137539E**+**03**	4.195931E+03	9.630819E+03	1.342648E+04	4.973065E+03	1.312238E+04	5.289105E+03	5.463600E+03
F11	4.504875E+02	2.057442E+02	1.486817E+03	2.039901E+04	6.783918E+03	2.113400E+03	1.674798E+02	**1.211800E**+**02**
F12	1.059572E+07	1.065257E+07	2.351800E+08	3.425085E+10	1.188365E+07	3.271057E+09	**2.437640E**+**05**	2.185300E+06
F13	9.126381E+03	**8.131464E**+**03**	1.525357E+06	1.085936E+10	1.751459E+05	1.605525E+07	9.078747E+03	9.899900E+03
F14	1.924467E+05	6.992403E+04	2.720629E+06	9.194658E+06	6.793255E+06	1.242455E+06	**3.347240E**+**04**	7.275900E+04
F15	9.303842E+03	**7.397000E**+**03**	1.298617E+05	3.010678E+09	8.930509E+04	3.905566E+05	1.110092E+04	1.708200E+04
F16	1.783052E+03	1.666879E+03	2.070131E+03	5.504998E+03	2.262889E+03	3.621052E+03	2.194528E+03	**1.399800E**+**03**
F17	1.180549E+03	1.210225E+03	1.420055E+03	4.938618E+03	1.630942E+03	2.183051E+03	1.698985E+03	**1.018100E**+**03**
F18	2.439978E+06	9.011033E+05	1.088339E+07	4.581218E+07	9.811066E+06	5.760661E+06	**1.475907E**+**05**	3.585100E+05
F19	1.689568E+04	**1.630419E**+**04**	8.573691E+04	9.926634E+08	2.151534E+04	2.510438E+05	1.973871E+04	3.257800E+04
F20	1.036089E+03	1.056018E+03	1.139860E+03	1.977251E+03	1.282583E+03	1.550433E+03	1.330054E+03	**7.105400E**+**02**
F21	3.426018E+02	**3.385712E**+**02**	5.461143E+02	1.028874E+03	4.600252E+02	7.358044E+02	4.786187E+02	3.446600E+02
F22	4.993967E+03	**4.963820E**+**03**	1.045070E+04	1.389557E+04	6.118906E+03	1.219152E+04	6.424262E+03	6.730300E+03
F23	5.906586E+02	5.983694E+02	8.118768E+02	1.658512E+03	6.996731E+02	1.098604E+03	7.541392E+02	**5.776900E**+**02**
F24	6.524504E+02	**6.309307E**+**02**	9.678781E+02	1.790192E+03	1.235010E+03	1.141504E+03	1.224072E+03	6.565000E+02
F25	5.963229E+02	5.840070E+02	1.012247E+03	1.671093E+04	5.816009E+02	3.323002E+03	5.584962E+02	**5.292800E**+**02**
F26	2.848024E+03	2.976773E+03	5.082305E+03	1.449128E+04	3.904785E+03	7.616505E+03	4.713186E+03	**2.827800E**+**03**
F27	**6.701314E**+**02**	6.858194E+02	8.658425E+02	2.625309E+03	9.062002E+02	1.384745E+03	9.657826E+02	6.745200E+02
F28	5.604839E+02	5.521116E+02	1.052934E+03	9.428823E+03	5.330770E+02	3.033216E+03	4.944299E+02	**4.877600E**+**02**
F29	1.045989E+03	1.033800E+03	1.568952E+03	8.428604E+03	1.566282E+03	3.344824E+03	1.557713E+03	**9.328100E**+**02**
F30	1.193221E+06	1.191038E+06	6.764392E+06	2.322438E+09	8.132439E+06	5.058966E+07	9.895572E+05	8.498800E+05
+\−\≈	19\9\2	20\10\0	30\0\0	30\0\0	26\4\0	30\0\0	19\10\1	

Significant values are in [bold].

**Table 13:** The comparison results of the algorithms with D = 50 on the standard deviation (SD).

	HS	IHS	GHS	SGHS	NGHS	IGHS	IMGHS	NIGHS
F1	5.784543E+06	1.694406E+05	8.702756E+08	2.122046E+10	1.283926E+05	3.509416E+09	7.311661E+03	**4.691000E**+**03**
F2	1.161688E+41	1.968422E+34	1.261509E+57	1.101077E+74	9.203381E+50	5.963579E+62	2.772672E−04	**1.826500E**−**04**
F3	4.378908E+03	4.964514E+03	1.456683E+04	2.074796E+04	4.634879E+04	1.194862E+04	**1.766647E**+**03**	2.215900E+03
F4	5.300559E+01	5.282569E+01	1.443285E+02	5.978049E+03	5.004677E+01	7.495487E+02	**3.697156E**+**01**	5.098500E+01
F5	2.551585E+01	**2.484258E**+**01**	3.719569E+01	3.776569E+01	4.602946E+01	2.545457E+01	5.333437E+01	3.339100E+01
F6	2.712357E−01	3.570043E−01	2.194519E+00	6.470537E+00	**3.711105E**−**02**	4.248862E+00	6.447571E−02	8.368300E−02
F7	**3.108205E**+**01**	4.735006E+01	4.286525E+01	3.318612E+02	4.712196E+01	4.186788E+01	5.775901E+01	3.361500E+01
F8	2.444904E+01	2.779855E+01	3.115182E+01	4.106315E+01	4.974214E+01	**2.336634E**+**01**	5.426560E+01	2.621700E+01
F9	**8.495546E**+**02**	9.556698E+02	2.448685E+03	3.969400E+03	2.674243E+03	3.087773E+03	3.753503E+03	1.928900E+03
F10	5.980151E+02	6.596048E+02	8.382727E+02	4.017444E+02	6.294540E+02	**3.604159E**+**02**	6.560682E+02	2.813900E+03
F11	2.750503E+02	1.676461E+02	6.764090E+02	3.417386E+03	5.416590E+03	5.966836E+02	4.880669E+01	**3.087700E**+**01**
F12	5.837899E+06	6.067770E+06	8.736327E+07	1.005923E+10	6.905793E+06	6.255513E+08	**1.469554E**+**05**	1.011700E+06
F13	9.942145E+03	**8.323002E**+**03**	7.246434E+05	3.971548E+09	2.339772E+05	1.080365E+07	9.402188E+03	1.056900E+04
F14	1.375306E+05	4.568101E+04	1.692122E+06	3.024779E+06	4.470063E+06	8.261358E+05	**2.044855E**+**04**	5.188300E+04
F15	6.964376E+03	6.807476E+03	8.874022E+04	9.973297E+08	6.597539E+04	5.522340E+05	6.980599E+03	**3.069500E**+**03**
F16	4.172558E+02	3.884086E+02	4.147065E+02	4.394834E+02	4.356693E+02	**3.106374E**+**02**	3.952837E+02	3.385700E+02
F17	3.406036E+02	2.964780E+02	3.078455E+02	5.751075E+02	3.480439E+02	**1.881319E**+**02**	3.687952E+02	2.317800E+02
F18	1.924174E+06	8.461552E+05	7.565492E+06	1.498629E+07	8.769992E+06	4.068220E+06	**7.090614E**+**04**	1.903100E+05
F19	1.310999E+04	**1.280621E**+**04**	4.346035E+04	4.970888E+08	1.607244E+04	5.614139E+05	1.302454E+04	1.438200E+04
F20	2.963722E+02	2.893793E+02	2.981384E+02	**1.745948E**+**02**	3.637333E+02	3.253751E+02	3.332243E+02	2.855200E+02
F21	**2.421513E**+**01**	2.524587E+01	3.711512E+01	3.817279E+01	5.076630E+01	2.829261E+01	4.866910E+01	2.488700E+01
F22	6.623105E+02	7.416622E+02	8.832326E+02	**3.746720E**+**02**	7.043077E+02	3.249091E+03	7.672180E+02	3.497500E+03
F23	**3.208150E**+**01**	4.170066E+01	3.813062E+01	9.517658E+01	4.566734E+01	3.840044E+01	7.195900E+01	3.376700E+01
F24	6.173000E+01	4.996106E+01	**2.570789E**+**01**	1.214874E+02	1.991362E+02	4.499440E+01	1.843306E+02	4.280300E+01
F25	2.881304E+01	3.186044E+01	1.320816E+02	3.780591E+03	**2.587490E**+**01**	4.493017E+02	4.132307E+01	3.868100E+01
F26	4.758123E+02	**3.220917E**+**02**	3.271611E+02	1.186622E+03	1.019869E+03	7.888649E+02	1.144875E+03	4.009100E+02
F27	7.018223E+01	8.427096E+01	7.579807E+01	2.155450E+02	9.987680E+01	1.111052E+02	1.387533E+02	**5.262100E**+**01**
F28	4.195256E+01	4.061944E+01	1.570587E+02	1.440829E+03	**2.615333E**+**01**	3.184364E+02	3.164146E+01	2.967700E+01
F29	2.596586E+02	**2.584116E**+**02**	3.393204E+02	1.593843E+03	3.603280E+02	3.099260E+02	3.582580E+02	2.657100E+02
F30	4.066627E+05	3.446764E+05	2.260862E+06	6.696556E+08	5.001140E+07	1.822205E+07	1.844713E+05	**1.372700E**+**05**
+\−\≈	18\8\4	20\10\0	26\4\0	27\3\0	24\4\2	24\5\1	20\9\1	

Significant values are in [bold].

In this experiment, NIGHS was compared with HS, IHS, GHS, SGHS, NGHS and IMGHS. As the NIGHS algorithm aims to improve the trust region of the IGHS algorithm to achieve a better balance between search and exploitation performance of the algorithm, the comparison with IGHS is particularly relevant. To this end, we first focus on comparing the experimental results of NIGHS and HS, IHS, GHS, SGHS, NGHS and IMGHS algorithms, then consider the comparison of experimental results of NIGHS and IGHS algorithms. In this way, the improvement of NIGHS can be highlighted as meaningful and progressive.

As for the HS, IHS, GHS, SGHS, NGHS, IMGHS algorithms, Tables 2, 3, 4 and 5 show that NIGHS algorithm is significantly better than them in the test results of these 30 benchmark functions with dimension size of130. Since the principle of the algorithm is based on the probability model, it is more convincing to use the average of 51 calculation results to explain. For the optimal values of 10 iterations, Table 4 reveals the following results:

1. Compared with the HS algorithm, NIGHS has 20 results better than HS in 30 test functions, and the remaining 10 results are slightly worse than HS.
2. Compared to the IHS algorithm, NIGHS is better than IHS in only 15 out of 30 functions, but the results of the remaining 15 functions are similar to that of IHS in 7 functions, and slightly better than IHS overall.
3. Compared with the GHS algorithm, NIGHS is superior to GHS in the test results of 22 functions, and significantly worse than GHS in F18.
4. Compared with SGHS algorithm, NIGHS is better than SGHS on 27 functions, slightly worse than SGHS on F21 and F24, and worse than SGHS on F14.
5. NIGHS is superior to both the NGHS and IMGHS algorithms on 28 functions and slightly inferior to them on F18, 25 and 28.

On the whole, the experimental calculation results of NIGHS at D = 10 are superior to the six algorithms. As the dimension increases, when D = 30, NIGHS algorithm outperforms HS, IHS, GHS, SGHS, NGHS and IMGHS on more functions. NIGHS outperforms them on 27, 25, 30, 30, 29, and 26, respectively. NIGHS outperforms 19, 20, 30, 30, 26, and 19 function results when D = 50. Although the comparison results decreased when D = 30, it was still better than the six algorithms on the whole. This is because in the early NIGHS computing, algorithm probability to use improved coupling operation, search in a wide range of avoid premature convergence, then high probability for stable trust region search and Gaussian fine tuning, speed up the convergence of the algorithm and improve the accuracy of convergence. Both IHS and NIGHS use the same dynamic tone width, so when the dimensions are low, the problem is less complex, and fine-tuning can be done to avoid falling into local optimal, with NIGHS only slightly better than IHS at D = 10. As the dimensions increase, NIGHS is significantly better than IHS when D = 30 and 50. This is because the improved coupling operation of NIGHS in the higher dimension, there is a higher randomness, to help the algorithm in the early stage of a large range of search, avoid falling into the local optimal.

The robust stability of an algorithm is often demonstrated by the standard deviation (SD) comparison of experimental results. From Tables 5, 9 and 13, we can see that when D = 10, NIGHS on 20, 15, 19, 23, 28, 29 functions is better than HS, IHS, GHS, SGHS, NGHS and IMGHS respectively. NIGHS outperforms 20, 20, 27, 27, 27, 24 functions in standard deviation when D = 30. When D = 50, NIGHS outperforms the standard deviation of 18, 20, 26, 27, 24, 20 functions. NIGHS performs poorly mainly on F10, F21 and F28 when D = 10. NIGHS performs poorly mainly on F10, F26, and F28 when D = 30. When D50, NIGHS performed poorly mainly on F10 and F22. Overall, NIGHS is significantly more robust than GHS, SGHS, NGHS and IMGHS, and better than HS. NIGHS and IHS are not much different in robustness at low dimensions, but as dimensions increase NIGHS still outperforms IHS.

The comparison of the best value and the worst value results of the 51 times of each algorithm is helpful to analyze the upper limit and lower limit of an optimization ability of the algorithm. We can see from Tables 2, 3, 6, 7, 10 and 11 that NIGHS is significantly superior to GHS,SGHS,NGHS and IMGHS, superior to HS and slightly superior to IHS when D = 10. NIGHS is significantly superior to all six algorithms when D = 30. At D = 50, the advantage of NIGHS declines, but it is still superior to the six algorithms. Searching in stable trust areas and dynamic Gaussian fine-tuning will help NIGHS 'algorithmic capabilities increase. The improved coupling operation can improve the ability of the algorithm to get rid of the local optimal and the lower limit of the algorithm's ability.

Now let us consider the comparison between IGHS and NIGHS. First of all, it can be seen from Tables 4, 8 and 12 that NIGHS has 28, 30, 30 function results better than IGHS on mean value when D = 10, 30 and 50. NIGHS outperformed IGHS on almost the entire test set. For robust stability, it can be found from Tables 5, 9 and 13 that NIGHS is superior to IGHS on 26,26 and 24 functions respectively when D = 10, 30 and 50 for standard deviation (SD). It can be proved that NIGHS is superior to IGHS in robustness in all dimensions. As for the upper limit and lower limit of the optimization ability of the algorithm, we compared the best value and the worst value of the results of 51 separate runs. As for the upper limit of optimization capability, we can see from Tables 2, 6 and 10 that NIGHS is superior to IGHS on 27,30, and 30 functions respectively when D = 10,30, and 50. As for the worst value, we can find from Tables 3, 7 and 11 that NIGHS is superior to IGHS in the calculation results of 28,29 and 29 functions respectively under the conditions of D = 10,30 and 50. According to the results of the algorithms on CEC2017 test function set, we can conclude that NIGHS 'optimization ability and robustness are much better than IGHS. This is because NIGHS has improved the coupling operation of different dimensions on IGHS algorithm framework, so that the original limited and lack of rationality of the operation has been improved, for the complex addition of displacement and rotation functions can also be applied. The original trust region search based on the worst harmony and the best harmony is improved. The mean harmony is used to construct the stable convergence of the stable trust region, and the dynamic Gauss fine-tuning strategy is added to improve the convergence speed and accuracy of the algorithm.

In sum, according to Tables 2, 3, 4, 5, 6, 7, 8, 9, 10, 11, 12 and 13, NIGHS has some obvious advantages over the comparison algorithms. It is superior to HS in terms of exploration ability and robustness and has better optimization performance than the IGHS in lower and high dimension.

### Further analysis

**Figure 8:**
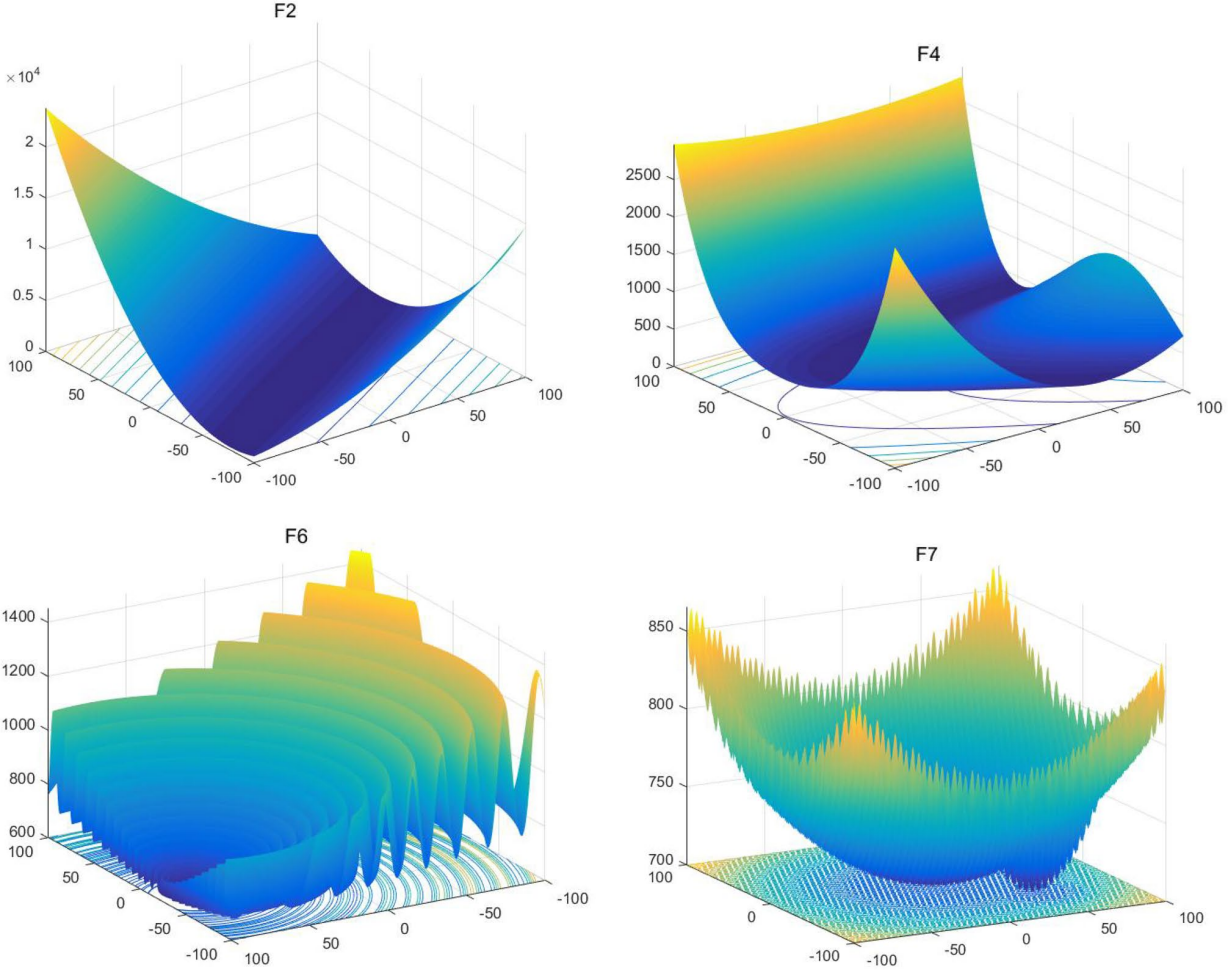
The graphs of F2, F4, F6 and F7 with dimension size of 2.

**Figure 9:**
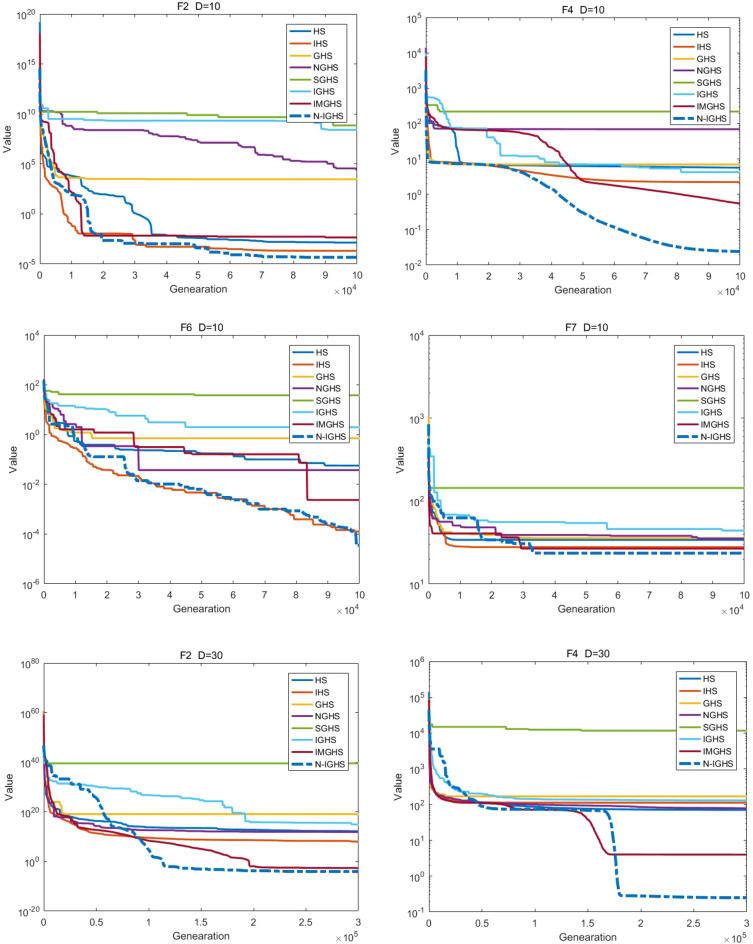

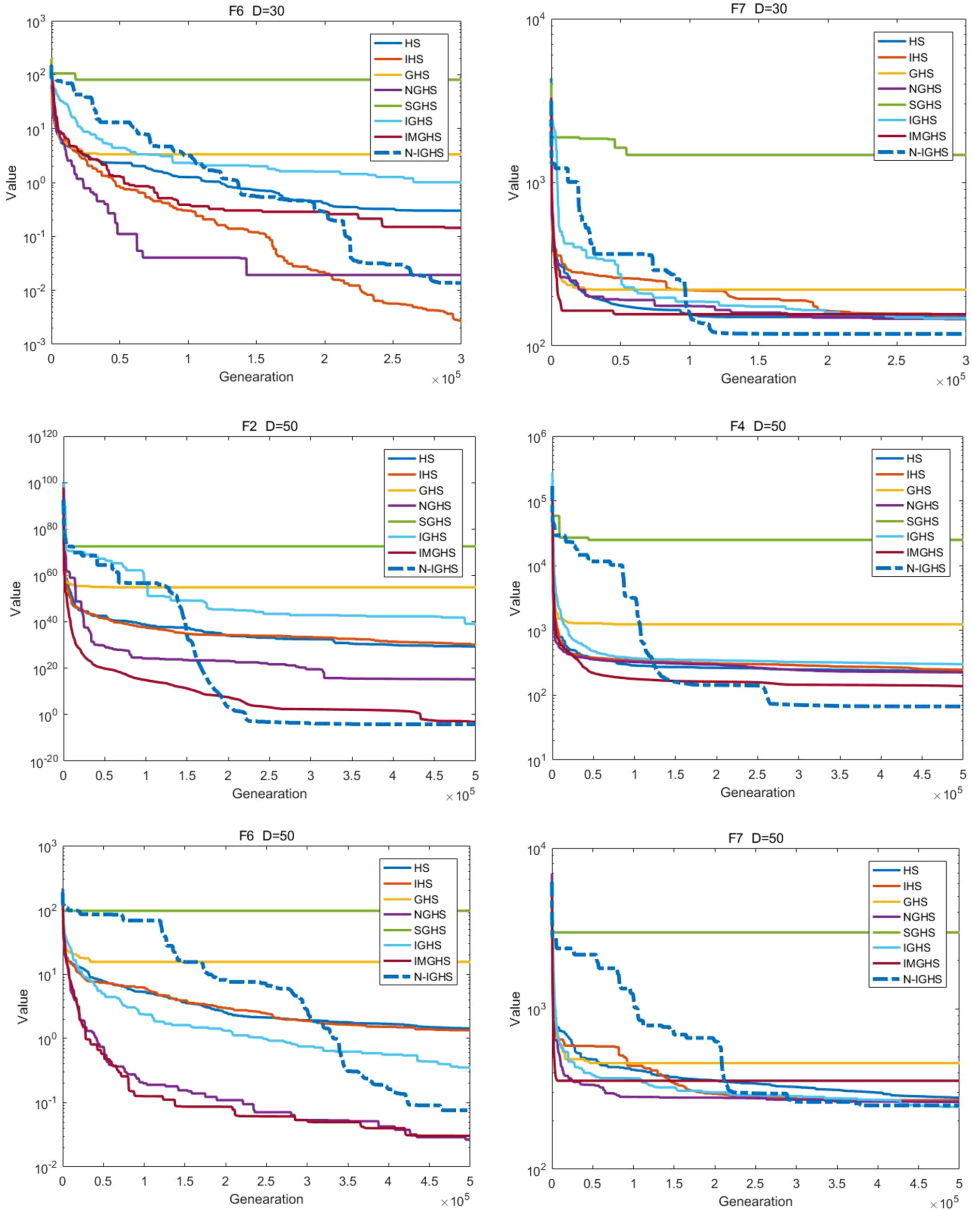
The iteration graphs of the comparison algorithms.

To analyze the search performance and convergence ability of the algorithms more intuitively, we selected one unimodal test functions (F2) and three Simple multimodal test functions (F4, F6 and F7), which are plotted the iteration graph of these eight algorithms. When the dimension is 2, the three-dimensional images of these four functions are shown in Fig. 8. When the variable dimensions are 10, 30 and 50 respectively, the calculated iteration graph are shown in Fig. 9, where the abscissa is the number of iterations and the ordinate is the fitness value of the optimal solution.

Figure 9 contains 12 graphs, which are the iteration graphs of the eight algorithms on F2, F4, F6 and F7 with dimension size of 10, 30, and 50, respectively. For the convergence analysis of the algorithm, we can prove it by analyzing the iterative graph of the historical optimal solution. In Fig. 9, we can see that since the first generation of the optimal solution, the fitness value of the historical optimal solution is converging to the corresponding global optimal solution as the iteration progresses. On F2, F4, F6 and F7, with continuous iteration, the historical optimal solution of NIGHS is constantly approaching the global optimal solution 0. This convergence behavior has been used in many literatures to prove that the algorithm has robust convergencey^78,79^.

From Fig. 8, we can see that there is no local optimal solution for the unimodal function F2. We can analyze the convergence ability of the algorithm by the calculation process of the algorithm on the function. According to the iterative calculation curves of F2 when D = 10, 30 and 50 in Fig. 9, we can find that the calculation result accuracy of NIGHS is the best among the eight algorithms in the final calculation results. IHS is slightly less accurate than NIGHS, but both algorithms are better than the other six. This is because both IHS and NIGHS employ a fine-tuning strategy with convergent BW. NIGHS is slightly better than IHS, the difference is that NIGHS uses the Gaussian fine-tuning, which compared to the probability of random fine-tuning in (0,1] of IHS, is more concentrated. Let NIGHS has higher convergence accuracy.

For a comparison of the running conditions in the simple multimodal functions F4, 6 and 7, we can see from Fig. 9 that the final calculation results of NIGHS on these three functions are superior to the other seven algorithms for D = 10,30 and 50. At the same time, we can observe that for local optimum of multimodal problems, HS, IHS, GHS, SGHS, NGHS, IGHS and IMGHS is convergence to an optimal value easily at the early stage of the calculation, and then has been stalled in the value. After that, most of the calculation are unable to find a better solution. Observe NIGHS performance in F7 in D = 10, 30, 50, NIGHS in the early computation compared to other algorithms to maintain a very slow search, and did not converge prematurely. NIGHS slowly convergence until running to the middle of the calculation. This is because NIGHS adjusts the HMCR and PAR on the move, taking into account the characteristics of the three strategies and adopting the right strategies at different times of computing. In the early stage of the calculation, NIGHS mainly adopts the improved coupling operation, so that the algorithm in the convergence domain for global search, and then gradually converted to local exploit, local convergence.

### The conclusions of experimental results

The purpose of this study is to improve the trust region and coupling operation of the IGHS algorithm, and to construct a new trust region with good stability and convergence, as well as a more reasonable coupling operation. By adaptively adjusting HMCR and PAR, the algorithm can use each strategy more reasonably at different stages, better balancing the search and development performance of the algorithm. Through the analysis of the iterative graphs of the selected four functions, it can be found that NIGHS can maintain a strong global search ability in the early stage of calculation when solving basic multi-peak problems, compared with other algorithms, suppressing the development ability and avoiding premature convergence into a local optimum. This indicates that NIGHS can adaptively adjust the exploration and development abilities during the search process to avoid premature convergence. At the same time, by testing the selected CEC2017 test function set and comparing the experimental data of the proposed NIGHS algorithm with the NGHS algorithm, which also constructs a trust region, it can be concluded that the convergence speed and calculation accuracy of NIGHS are better than NGHS in most problems. This indicates that using dynamic Gaussian fine-tuning in a stable trust region can accelerate convergence speed and improve optimization accuracy. Based on the above, it can be concluded that although the NIGHS algorithm proposed in this paper may not be the best choice for all problem considerations, it can basically meet our requirements. Overall, for single-objective unconstrained optimization problems, NIGHS is a relatively good choice.

## Conclusions

In this paper, we proposed a novel intelligent global harmony search algorithm, which improves upon the trust region of the previous algorithm by considering the best and worst values to provide a more stable trust region with better convergence. Additionally, we introduced modifications to the simple coupling operation of IGHS based on the linear proportional relationship between different dimensions. Our proposed NIGHS algorithm was tested and evaluated using the CEC2017 test function set, and compared with several other algorithms including the basic version of harmony search, improved harmony search algorithm, geometric harmony search algorithm, novel global harmony search algorithm, self-adaptive global best harmony search algorithm, intelligent global harmony search algorithm, and intersect mutation global harmony search algorithm.

Our experimental results demonstrate that the proposed algorithm outperforms the HS and IGHS algorithms in terms of solution accuracy and efficiency, and has robust convergence. This improved efficiency is attributed to our stable trust region and improved coupling operation, which strike a balance between exploration and exploitation performance of the algorithm. Additionally, the dynamic Gauss fine-tuning improves the solution accuracy of the algorithm. We believe that our research has significance and rationality in the application of the coupling operation, and that normalization of the variables of each dimension, unifying the values of each dimension, or reducing the difference between the values of each dimension can help to establish the mathematical model of actual problems.

Even though different solutions may have varying physical meanings, we posit that effective solutions can still be obtained by searching the data space as a spatial process, and that different dimensional boundaries also have common parts. If the difference between different dimensions is still large after normalization, this can be addressed by reducing PAR. Furthermore, our proposed NIGHS algorithm is easy to implement and provides a good choice for solving complex global optimization problems.

In the future research, we will focus on further practical engineering applications of our proposed algorithm in fields such as power systems, image processing, network optimization, and high-order SNP interaction detection. This can shed more light on the algorithm and its potential for solving real-world problems.

## Data Availability

The data used to support the findings of this study are available from the corresponding author upon request.

## References

[1] Lee KS, Geem ZW, Lee SH, Bae KW (2005). The harmony search heuristic algorithm for discrete structural optimization. Eng. Optim..

[2] Geem ZW (2006). Optimal cost design of water distribution networks using harmony search. Eng. Optim..

[3] Geem ZW, Lee KS, Park Y (2005). Application of harmony search to vehicle routing. Am. J. Appl. Sci..

[4] Metawaa N, Hassana MK, Elhoseny M (2017). Genetic algorithm based model for optimizing bank lending decisions. Expert Syst. Appl..

[5] Chen KH, Chen LF, Su CT (2014). A new particle swarm feature selection method for classification. J. Intell. Inf. Syst..

[6] Mirjalili S, Lewis A (2016). The Whale optimization algorithm. Adv. Eng. Softw..

[7] Alsattar HA, Zaidan AA, Zaidan BB (2020). Novel meta-heuristic bald eagle search optimization algorithm. Artif. Intell. Rev. Int. Sci. Eng. J..

[8] Kumar V, Chhabra JK, Kumar D (2012). Effect of harmony search parameters’ variation in clustering. Procedia Technol..

[9] Nancy M, Stephen SEA (2021). A comprehensive review on harmony search algorithm. Ann. Roman. Soc. Cell Biol..

[10] Liu L, Zhou H (2013). Hybridization of harmony search with variable neighborhood search for restrictive single-machine earliness/tardiness problem. Inf. Sci..

[11] Wang L, Pan Q-K, Tasgetiren MF (2011). A hybrid harmony search algorithm for the blocking permutation flow shop scheduling problem. Comput. Ind. Eng..

[12] Yuan Y, Xu H, Yang J (2013). A hybrid harmony search algorithm for the flexible job shop scheduling problem. Appl. Soft Comput..

[13] Alatas B (2010). Chaotic harmony search algorithms. Appl. Math. Comput..

[14] Al-Betar MA, Doush IA, Khader AT, Awadallah MA (2012). Novel selection schemes for harmony search. Appl. Math. Comput..

[15] Ashrafi SM, Dariane AB (2013). Performance evaluation of an improved harmony search algorithm for numerical optimization: Melody search (MS). Eng. Appl. Artif. Intell..

[16] Chen J, Pan Q-k, Li J-q (2012). Harmony search algorithm with dynamic control parameters. Appl. Math. Comput..

[17] Cobos C, Estupiñan D, Pérez J (2011). GHS + LEM: Global-best harmony search using learnable evolution models. Appl. Math. Comput..

[18] Geem ZW, Sim K-B (2010). Parameter-setting-free harmony search algorithm. Appl. Math. Comput..

[19] Omran MGH, Mahdavi M (2008). Global-best harmony search. Appl. Math. Comput..

[20] Pan Q-K, Suganthan PN, Tasgetiren MF, Liang JJ (2010). A self-adaptive global best harmony search algorithm for continuous optimization problems. Appl. Math. Comput..

[21] Wang C-M, Huang Y-F (2010). Self-adaptive harmony search algorithm for optimization. Expert Syst. Appl..

[22] Wang G, Guo L (2013). A novel hybrid bat algorithm with harmony search for global numerical optimization. J. Appl. Math..

[23] Wu B, Qian C, Ni W, Fan S (2012). Hybrid harmony search and artificial bee colony algorithm for global optimization problems. Comput. Math. Appl..

[24] Yadav P, Kumar R, Panda SK, Chang CS (2012). An intelligent tuned harmony search algorithm for optimisation. Inf. Sci..

[25] Zou D, Gao L, Wu J, Li S (2010). Novel global harmony search algorithm for unconstrained problems. Neurocomputing.

[26] Kulluk S, Ozbakir L, Baykasoglu A (2011). Self-adaptive global best harmony search algorithm for training neural networks. Procedia Comput. Sci..

[27] Geem ZW (2009). Particle-swarm harmony search for water network design. Eng. Optim..

[28] Zou D, Gao L, Li S, Wu J (2011). Solving 0–1 knapsack problem by a novel global harmony search algorithm. Appl. Soft Comput..

[29] Sarkhel R, Das N, Saha AK, Nasipuri M (2018). An improved harmony search algorithm embedded with a novel piecewise opposition based learning algorithm. Eng. Appl. Artif. Intell..

[30] Mahdavi M, Fesanghary M, Damangir E (2007). An improved harmony search algorithm for solving optimization problems. Appl. Math. Comput..

[31] Khalili M, Kharrat R, Salahshoor K, Sefat MH (2014). Global dynamic harmony search algorithm: GDHS. Appl. Math. Comput..

[32] Zhu Q, Tang X, Li Y, Yeboah MO (2020). An improved differential-based harmony search algorithm with linear dynamic domain. Knowl.-Based Syst..

[33] Li S, Zhang D, Shao Z, Tang H (2020). Information feedback self-adaptive harmony search algorithm for the bovine cortical bone vibration-assisted drilling optimization. Measurement.

[34] Li HC, Zhou KQ, Mo LP, Zain AM, Qin F (2020). Weighted fuzzy production rule extraction using modified harmony search algorithm and BP neural network framework. IEEE Access.

[35] Mahmoudi SM, Rad MM, Ochbelagh DR (2021). Hybrid of the fuzzy logic controller with the harmony search algorithm to PWR in-core fuel management optimization. Nuclear Eng. Technol..

[36] Loor A, Bidgoli M, Hamid M (2021). Optimization and buckling of rupture building beams reinforced by steel fibers on the basis of adaptive improved harmony search-harmonic differential quadrature methods. Case Stud. Constr. Mater..

[37] Cui Y, Dong W, Hu D, Liu H (2022). The application of improved harmony search algorithm to multi-UAV task assignment. Electronics.

[38] Tsakirakis E, Marinaki M, Marinakis Y, Matsatsinis N (2019). A similarity hybrid harmony search algorithm for the team orienteering problem. Appl. Soft Comput..

[39] Li Z, Zou D, Kong Z (2019). A harmony search variant and a useful constraint handling method for the dynamic economic emission dispatch problems considering transmission loss. Eng. Appl. Artif. Intell..

[40] Boryczka U, Szwarc K (2019). The harmony search algorithm with additional improvement of harmony memory for asymmetric traveling salesman problem. Expert Syst. Appl..

[41] Doush IA, Al-Betar MA, Awadallah MA (2019). Flow shop scheduling with blocking using modified harmony search algorithm with neighboring heuristics methods. Appl. Soft Comput..

[42] Liu C, Abdulkareem SS, Rezvani A, Samad S, Aljojo N, Foong LK, Nishihara K (2020). Stochastic scheduling of a renewable-based microgrid in the presence of electric vehicles using modified harmony search algorithm with control policies. Sustain. Cities Soc..

[43] Wang M, Zhang T, Wang P, Chen X (2020). An improved harmony search algorithm for solving day-ahead dispatch optimization problems of integrated energy systems considering time-series constraints. Energy Build..

[44] Zhu Q, Tang X (2021). An ameliorated harmony search algorithm with hybrid convergence mechanism. IEEE Access.

[45] Pan Z, Zhang LW, Liew KM (2021). Adaptive surrogate-based harmony search algorithm for design optimization of variable stiffness composite materials. Comput. Methods Appl. Mech. Eng..

[46] Li X, Li X, Yang G (2022). A novelty harmony search algorithm of image segmentation for multilevel thresholding using learning experience and search space constraints. Multimedia Tools Appl..

[47] Sörensen K (2015). Metaheuristics—the metaphor exposed. Int. Trans. Oper. Res..

[48] Fesanghary M, Mahdavi M, Minary-Jolandan M, Alizadeh Y (2008). Hybridizing harmony search algorithm with sequential quadratic programming for engineering optimization problems. Comput. Methods Appl. Mech. Eng..

[49] Wang L, Li LP (2012). A coevolutionary differential evolution with harmony search for reliability–redundancy optimization. Expert Syst. Appl..

[50] Kayabekir AE, Toklu YC, Bekdaş G, Nigdeli SM, Yücel M, Geem ZW (2020). A novel hybrid harmony search approach for the analysis of plane stress systems via total potential optimization. Appl. Sci..

[51] Shaikh TA, Ali R (2020). An intelligent healthcare system for optimized breast cancer diagnosis using harmony search and simulated annealing (HS-SA) algorithm. Inform. Med. Unlocked.

[52] Zhang, Y., Li, J. & Li, L. (2021). An improved clustering-based harmony search algorithm (IC-HS). In Proceedings of SAI Intelligent Systems Conference (pp. 115–124).

[53] Radman A (2021). Combination of BESO and harmony search for topology optimization of microstructures for materials. Appl. Math. Model..

[54] Gong J, Zhang Z, Liu J, Guan C, Liu S (2021). Hybrid algorithm of harmony search for dynamic parallel row ordering problem. J. Manuf. Syst..

[55] Amini F, Ghaderi P (2013). Hybridization of harmony search and ant colony optimization for optimal locating of structural dampers. Appl Soft Comput.

[56] Gheisarnejad M (2018). An effective hybrid harmony search and cuckoo optimization algorithm based fuzzy PID controller for load frequency control. Appl Soft Comput.

[57] Li G, Zeng B, Liao W, Li X, Gao L (2018). A new AGV scheduling algorithm based on harmony search for material transfer in a real-world manufacturing system. Adv. Mech. Eng..

[58] Szwarc K, Boryczka U (2022). A novel approach to the Orienteering Problem based on the Harmony Search algorithm. PLoS ONE.

[59] Wang H, Xu H, Gao XZ, Zhao Z, Huang J (2021). Arrangement optimization of a novel three dimensional multiphase flow imaging device employing modified harmony search algorithm. Eng. Appl. Artif. Intell..

[60] Huang YF, Chen PH (2020). Fake news detection using an ensemble learning model based on self-adaptive harmony search algorithms. Expert Syst. Appl..

[61] Yong L (2022). Novel global harmony search algorithm for general linear complementarity problem. Axioms.

[62] Jeddi B, Vahidinasab V, Ramezanpour P, Aghaei J, Shafie-khah M, Catalão JP (2019). Robust optimization framework for dynamic distributed energy resources planning in distribution networks. Int. J. Electr. Power Energy Syst..

[63] Maleki A, Nazari MA, Pourfayaz F (2020). Harmony search optimization for optimum sizing of hybrid solar schemes based on battery storage unit. Energy Rep..

[64] Dash R (2021). An adaptive harmony search approach for gene selection and classification of high dimensional medical data. J. King Saud Univ.-Comput. Inf. Sci..

[65] Botella Langa A, Choi Y-G, Kim K-S, Jang D-W (2022). Application of the harmony search algorithm for optimization of WDN and assessment of pipe deterioration. Appl. Sci..

[66] Liu J, Mei L, Maleki A, Ghasempour R, Pourfayaz FA (2022). Global dynamic harmony search for optimization of a hybrid photovoltaic-battery scheme: Impact of type of solar panels. Sustainability.

[67] Hadwan M (2022). Annealing harmony search algorithm to solve the nurse rostering problem. Comput. Mater. Continua..

[68] Tuo S, Li C, Liu F (2022). MTHSA-DHEI: Multitasking harmony search algorithm for detecting high-order SNP epistatic interactions. Complex Intell. Syst..

[69] Tuo S, Liu H, Chen H (2020). Multipopulation harmony search algorithm for the detection of high-order SNP interactions. Bioinformatics.

[70] Tuo S, Geem ZW, Yoon JH (2020). A new method for analyzing the performance of the harmony search algorithm. Mathematics.

[71] Valian E, Tavakoli S, Mohanna S (2014). An intelligent global harmony search approach to continuous optimization problems. Appl. Math. Comput..

[72] Geem ZW, Kim JH, Loganathan GV (2001). A new heuristic optimization algorithm: Harmony search. SIMULATION.

[73] Omran MGH, Mahdavi M (2008). Global-best harmony search. Appl. Math. Comput..

[74] Zou D, Gao L, Li S, Wu J, Wang X (2010). A novel global harmony search algorithm for task assignment problem. J. Syst. Softw..

[75] Gholami J, Ghany KKA, Zawbaa HM (2021). A novel global harmony search algorithm for solving numerical optimizations. Soft Comput..

[76] Awad, N., Ali, M., Liang, J., Qu, B. & Suganthan, P. Problem Definitions and Evaluation Criteria for the CEC 2017 Special Session and Competition on Single Objective Real-Parameter Numerical Optimization, Tech. Rep.,2016.

[77] Wolpert DH, Macready WG (1997). No free lunch theorems for optimization. IEEE Trans. Evol. Comput..

[78] Mirjalili S, Mirjalili SM, Lewis A (2014). Grey wolf optimizer. Adv. Eng. Softw..

[79] Faramarzi A, Heidarinejad M, Stephens B, Mirjalili S (2020). Equilibrium optimizer: A novel optimization algorithm. Knowl.-Based Syst..

